# Lower limb muscle strength and balance in older adults with a distal radius fracture: a systematic review

**DOI:** 10.1186/s12891-023-06711-4

**Published:** 2023-09-18

**Authors:** Colin Forde, Philippa JA Nicolson, Charlotte Vye, Jessica CH Pun, Warren Sheehan, Matthew L Costa, Sarah E Lamb, David J Keene

**Affiliations:** 1 Nuffield Department of Orthopaedics, Rheumatology and Musculoskeletal Sciences, University of Oxford, Oxford, UK; 2 Therapies Department, Royal United Hospitals NHS Foundation Trust, Bath, UK; 3 Institute of Child Health, University College London, London, UK; 4 Physiotherapy Department, Oxford University Hospitals NHS Foundation Trust, Oxford, UK; 5 Exeter Medical School, University of Exeter, Exeter, UK

**Keywords:** Falls, Balance, Muscle strength, Rehabilitation, Fragility fracture, Wrist fracture, Colles fracture

## Abstract

**Background:**

Distal radius fractures are common fractures in older adults and associated with increased risk of future functional decline and hip fracture. Whether lower limb muscle strength and balance are impaired in this patient population is uncertain. To help inform rehabilitation requirements, this systematic review aimed to compare lower limb muscle strength and balance between older adults with a distal radius fracture with matched controls, and to synthesise lower limb muscle strength and balance outcomes in older adults with a distal radius fracture.

**Methods:**

We searched Embase, MEDLINE, and CINAHL (1990 to 25 May 2022) for randomised and non-randomised controlled clinical trials and observational studies that measured lower limb muscle strength and/or balance using instrumented measurements or validated tests, in adults aged ≥ 50 years enrolled within one year after distal radius fracture. We appraised included observational studies using a modified Newcastle-Ottawa Scale and included randomised controlled trials using the Cochrane risk-of-bias tool. Due to the clinical and methodological heterogeneity in included studies, we synthesised results narratively in tables and text.

**Results:**

Nineteen studies (10 case-control studies, five case series, and four randomised controlled trials) of variable methodological quality and including 1835 participants (96% women, mean age 55–73 years, median sample size 82) were included. Twelve included studies (63%) assessed strength using 10 different methods with knee extension strength most commonly assessed (6/12 (50%) studies). Five included case-control studies (50%) assessed lower limb strength. Cases demonstrated impaired strength during functional tests (two studies), but knee extension strength assessment findings were conflicting (three studies). Eighteen included studies (95%) assessed balance using 14 different methods. Single leg balance was most commonly assessed (6/18 (33%) studies). All case-control studies assessed balance with inconsistent findings.

**Conclusion:**

Compared to controls, there is some evidence that older adults with a distal radius fracture have impaired lower limb muscle strength and balance. A cautious interpretation is required due to inconsistent findings across studies and/or outcome measures. Heterogeneity in control participants’ characteristics, study design, study quality, and assessment methods limited synthesis of results. Robust case-control and/or prospective observational studies are needed.

**Registration:**

International prospective register of systematic reviews (date of registration: 02 July 2020, registration identifier: CRD42020196274).

**Supplementary Information:**

The online version contains supplementary material available at 10.1186/s12891-023-06711-4.

## Introduction

Distal radius fractures are common, representing 18% of all fractures [1]. The economic burden of this injury is significant. In 2017, upper limb fractures incurred 33% of healthcare costs for fragility fractures in six European countries, around £1.7 billion [2]. After 50 years of age, women are nearly five times more likely than men to fracture their distal radius [3]. After this injury, postmenopausal women have a 48% increase in the odds of functional decline [4] and an approximately two-fold increase in three-year future hip fracture risk [5]. Given the high incidence of distal radius fractures, there are important implications for the healthcare system and wider society.

Most distal radius fractures in older adults occur following a fall from standing height onto an outstretched hand [6]. Clinical guidelines recommend that older adults attending healthcare services for a fall-related injury should undergo a falls-risk assessment which may include an assessment of balance and muscle strength [7]. If people are identified as being at increased risk of falls, muscle strengthening and balance exercises are recommended as part of an individualised multifactorial intervention [7]. This approach is supported by a systematic review which found high-certainty evidence that balance and functional exercises alone, or in addition to muscle strengthening exercises, reduce falls in community-dwelling older adults [8]. Previous literature has also recommended rehabilitation for older adults after distal radius fracture to reduce the risk of future fractures, falls, and functional decline [4, 6]. Despite this, interventions in trials evaluating rehabilitation for people with a distal radius fracture have focused on upper limb impairments, with limited prescription of balance and lower limb muscle strengthening exercises [9, 10].

However, older adults with a distal radius fracture are typically high functioning pre-injury [4] and younger than other fragility fracture populations [11]. Whether lower limb muscle strength and balance are impaired in this patient population compared to age- and sex/gender-matched controls is uncertain and to our knowledge has not been evaluated in a systematic review. To help inform whether rehabilitation targeting these modifiable variables is required, this systematic review aimed to: (1) compare lower limb muscle strength and balance between adults aged ≥ 50 years with a distal radius fracture and age- and sex/gender-matched controls, and (2) synthesise lower limb muscle strength and balance outcomes in adults aged ≥ 50 years with a distal radius fracture.

## Methods

The systematic review protocol was prospectively registered on the international prospective register of systematic reviews (PROSPERO, registration identifier: CRD42020196274). This report was written following preferred reporting items for systematic review and meta-analyses (PRISMA) guidelines [12].

### Search strategy

We searched Embase, MEDLINE, and CINAHL electronic databases on 18 June 2020, and updated this search on 25 May 2022. Search results were limited to studies published since 1990 so that included participants were more reflective of the current older adult population. No other search limitations were applied. The full search strategy for each database is presented in Additional file 1. To identify additional potentially eligible studies, we searched the reference lists of included studies and relevant systematic reviews.

### Eligibility criteria

We included randomised and non-randomised controlled clinical trials, and observational studies except single-patient case reports. Published reports, including abstracts, were eligible. Participants had to be women or men aged ≥ 50 years (or ≥ 90% of the sample was comprised of participants aged ≥ 50 years) enrolled within one year after a distal radius fracture treated surgically or non-surgically. Participants had to be aged ≥ 50 years because this is the most commonly affected age group [3]. Enrolment within one year after fracture aimed to limit the influence of advancing age and/or other disease processes on lower limb muscle strength and balance outcomes. Lower limb muscle strength or balance had to be assessed using instrumented measurements or validated physical performance tests. Only English, or non-English language studies adequately translated with Google Translate, were eligible. There was no limitation on study setting or follow-up duration.

During full-text screening, it became apparent that some studies included participants aged < 50 years, but participants’ mean age minus two standard deviations indicated ≥ 90% were aged ≥ 50 years [13–16]. Several studies did not specify if participants were enrolled within one year after distal radius fracture, instead they reported participants’ duration after distal radius fracture as a range, for example 6–24 months [16–20]. One study included a mixture of participants enrolled within, and more than, one year after distal radius fracture [21]. In all these instances we elected to be inclusive. Further details on these studies and reasons for inclusion are available in Additional file 2.

### Study selection

After duplicate removal, two reviewers (PJAN, CV, CF, or DJK) independently screened study titles and abstracts (where available) for eligibility. One reviewer (DJK) resolved any disagreements. Full texts of potentially eligible studies were then independently screened for eligibility by two reviewers (PJAN, DJK, or CF) who discussed any disagreements until consensus was reached.

### Data extraction

The following data was independently extracted by one reviewer (WS, CV, JCHP, or CF) and checked by another (PJAN, CF, CV): report characteristics (authors and publication year); study characteristics (design, location, eligibility criteria, intervention details, follow-up timepoints); participants’ characteristics (age, sex/gender, injury characteristics, duration from distal radius fracture, falls history); number of participants enrolled and that underwent lower limb muscle strength and/or balance assessment; and lower limb muscle strength and/or balance assessment method and results. Mistakes in extracted data were corrected by the second reviewer when an extraction error was clear. A third reviewer was consulted if there were uncertainties. We did not attempt to obtain missing data from the authors of included studies.

To identify multiple reports of the same study we compared the locations, authors’ names, participants’ characteristics, and duration of reports. We considered all reports of studies and combined data from multiple reports, where possible.

### Quality assessments

Quality assessments were completed at the outcome level (lower limb muscle strength and/or balance). Case-control studies and case series were assessed using a modified Newcastle-Ottawa Scale [22]. We modified the scale by removing the question ‘same method of ascertainment for cases and controls’ for case-control studies as this question does not apply to this review. For case series, we removed the questions on selection of the non-exposed cohort and comparability of cohorts, as these do not apply to case series. Therefore, case-control studies could score a maximum of eight stars and case series a maximum of five stars. For this review, we defined a case series as a single-group study that only included participants with a distal radius fracture. Randomised controlled trials (RCTs) were assessed using the Cochrane risk-of-bias tool [23] under the following domains: ‘selection bias’, ‘performance bias’, ‘detection bias’, ‘attrition bias’, ‘reporting bias’, and ‘other bias’.

Two reviewers (CF, PJAN, or DJK) independently appraised included studies. One reviewer (DJK) resolved disagreements. We did not make an overall risk-of-bias judgement across all included studies because different appraisal tools were used and included studies varied in design.

### Analysis

There was high clinical and methodological heterogeneity between included studies, so we did not complete a meta-analysis. Instead, we synthesised results narratively in tables and text.

We planned to group outcomes into short-term (≤ 4 months after fracture), medium-term (> 4–8 months after fracture) and long-term (> 8 months after fracture). However, the duration from distal radius fracture to strength and/or balance assessment was often not reported or unclear, so this was not completed.

To aid comparison of results between studies, we converted outcome data reported in pounds into kilograms (by multiplying pounds by 0.453592), and inches into centimetres (by multiplying inches by 2.54).

### Changes from protocol

We did not plan to compare lower limb muscle strength and balance between adults aged ≥ 50 years with a distal radius fracture with age- and sex/gender-matched controls, but due to the high number of included case-control studies and the potential clinical relevance of this comparison, this was completed.

Impaired lower limb muscle strength and balance are associated with increased falls risk in older adults [24, 25]. Therefore, we extracted participants’ falls history, though this was not pre-planned, to better assess the characteristics of participants in included studies.

We planned to include walking and gait assessments within the balance outcomes category, however it was deemed during study selection that a narrower focus on balance-specific measures was indicated due to the considerable heterogeneity in outcome assessment methods.

To facilitate comparison of results across studies, we analysed results by lower limb muscle strength and balance assessment method. This was not pre-planned. No other subgroup or sensitivity analyses were planned or conducted.

## Results

**Fig. 1:**
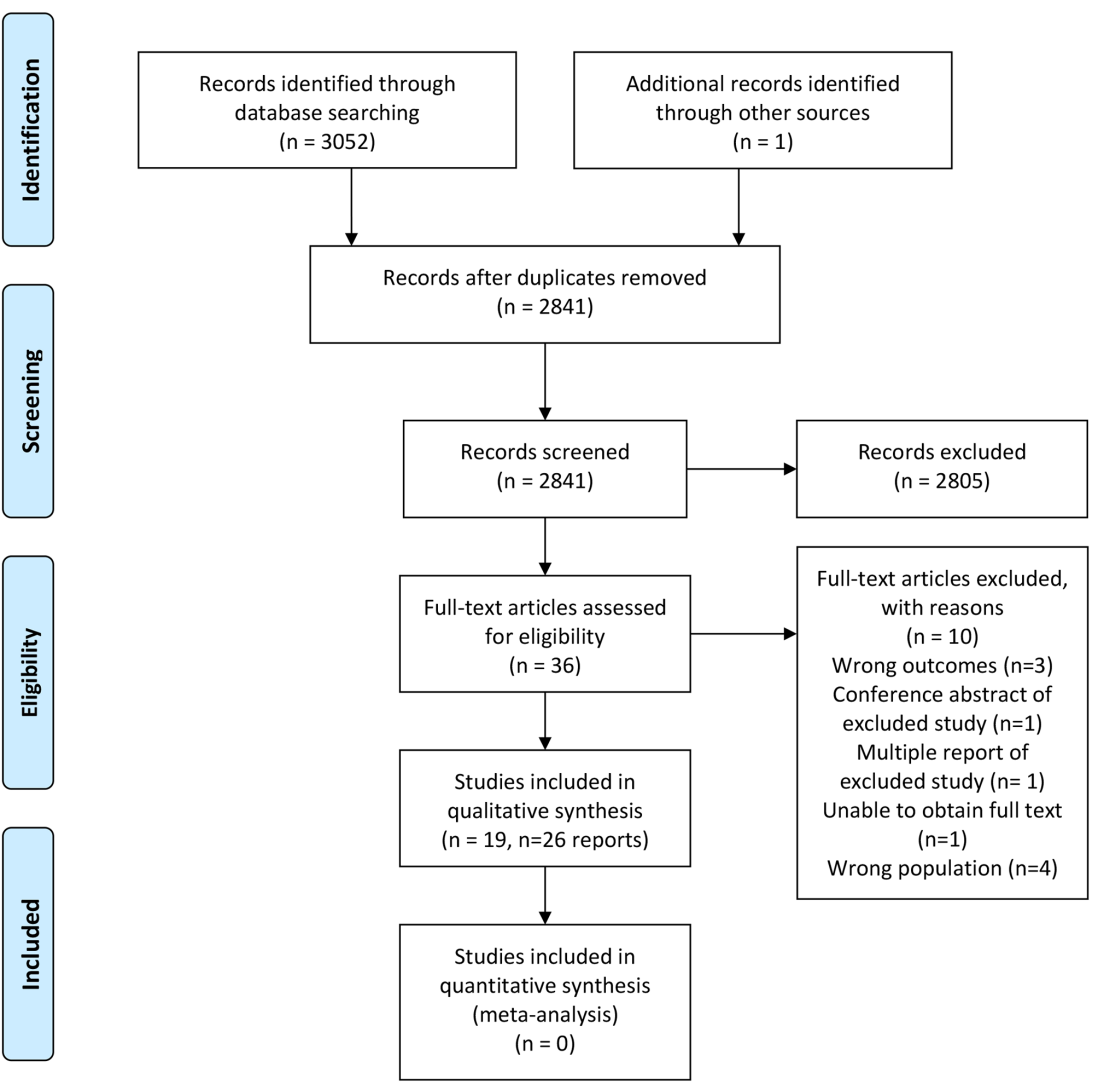
PRISMA flow diagram of systematic review search and screening process

The search strategy identified 3053 records. After duplicate removal, 2841 titles and abstracts were screened for eligibility. Thirty-six full-text reports underwent eligibility assessment. Twenty-six reports of 19 studies were subsequently included in the review. The systematic review search and screening process is shown in Fig. 1.

### Study characteristics

Of the 19 included studies, 10 (53%) were case-control studies, five (26%) were case series, and four (21%) were RCTs. Included studies were conducted in 11 different countries (Canada (four studies); Sweden and Japan (three studies); USA (two studies); China, Iceland, Iran, Ireland, Norway, South Korea, and UK (one study)). Fourteen (74%) studies were published since 2012. In total, studies included 1301 participants with a distal radius fracture (1835 participants including controls without a distal radius fracture). Almost all participants with a distal radius fracture were women (n = 1153/1211 (95%), data available in 18 studies). Twelve (63%) studies included women only. Participants’ mean age ranged from 55 to 73 years and the median sample size was 82 (interquartile range 66 to 103). Detailed characteristics of included studies are presented in Table 1. Additional file 3 describes the instrumented balance assessment procedures and scoring methods because these assessments are not commonly used in clinical settings.

**Table 1:** Characteristics of included studies

Study	Year	Country	Sample Size	Inclusion criteria	^†^Falls history	Mean age (SD); % women/ females (n)	Intervention/ rehabilitation	Control	Main outcome(s) reported
**Case-control studies**									
Cho et al., [26]	2014	South Korea	80 Cases: 40 Con: 40	Cases: Post-menopausal women, aged > 50 years, surgically or non-surgically treated DRF following a fall 6 months before enrolment Con: Age-matched post-menopausal women with a unilateral upper-limb condition	Cases: NR Con: 0 in previous 2 years	Cases: 60 (6.9) Con: 60.5 (7.4) 100% women (80)	NR	NR	5 STS Balance component of Short Physical Performance Battery
Crockett et al., [17]	2018	Canada	77 Cases: 32 Con: 45	Cases: Post-menopausal women aged ≥ 50 years, 6–24 months after DRF following fall from standing height Con: As above but no DRF since age 35 years	NR	Cases: 64 (8.4) Con: 62.5 (8.7) 100% women (77)	Received an unspecified home exercise programme after DRF	N/A	30s STS BBS Backwards tandem walk
Edwards et al., [18]	2006	USA	50 Cases: 26 Con: 24	Cases: Women aged > 50 years, community dwelling, independently mobile, DRF following a fall in past 6–24 months, low BMD Con: As above, fall in previous 2 years but no fracture	All participants averaged 1 fall in previous 12 months, data per treatment group NR	Cases: 70 (10.2) Con: 71.1 (7.2) 100% women (50)	NR	NR	Knee extension strength BBS
Fujita et al., [14]	2019	Japan	256 Cases: 128 Con: 128	Cases: Post-menopausal women, aged > 40 years, DRF was first fragility fracture following a fall from standing height or below, surgically treated Con: Age-matched post-menopausal women with no previous fragility fractures	NR	Cases: 66.9 (9.3) Con: 65.4 (9.5) 100% women (256)	Unspecified usual care physiotherapy for DRF	N/A	Functional Reach Test
Hakestad et al., [21]	2014	Norway	54 Cases: 36 Con: 18	Cases: Post-menopausal women aged > 50 years, osteopenia, healed DRF ≤ 2 years old Con: Healthy post-menopausal women aged > 50 years, no osteoporosis or previous fractures, matched to cases on age (± 5 years) height, weight, and BMI	NR	^‡^Cases: 59.1 (range 54 to 65) Con: 58.5 (range 51 to 65) 100% women	^§^UC	N/A	Knee extension strength Four Square Step Test
Louer et al., [19]	2016	USA	46 Cases: 23 Con: 23	Cases: Aged ≥ 65 years, 6–24 months after DRF following a fall from standing height, speaks English Con: Age- and sex-matched people, no previous fragility fracture	In previous 1 year Cases: median 1 (SD 2.2) Con: median 0 (SD 1.3)	Cases: 72.7 (5.2) Con: 72 (5.1) 91% female (42)	2/23 cases received prior or current balance therapy	1/23 control participants received prior or current balance therapy	Dynamic postural stability
O’Reilly et al., [28]	2013	Ireland	82 Cases: 41 Con: 41	Cases: Aged 55–80 years, low trauma DRF following a fall, community dwelling, independently mobile with or without mobility aid, speaks English Con: As above and no DRF or falls in previous 1 year	In previous 1 year Cases: 15/41 (37%) had > 1 fall Con: 0	Cases: 67.3 (7) Con: 68.5 (5.7) 90% women (74)	^¶^1 physiotherapy session of flexibility exercises and advice. 10 (24%) cases received balance exercises and additional physiotherapy sessions. 11 (27%) cases referred to community physiotherapy	^¶^2/41 (5%) controls received balance exercises and physiotherapy sessions. 2/41 (5%) controls were referred to community physiotherapy	Knee extension strength Knee flexion strength Ankle dorsiflexion strength MCToSIoB
Ringsberg et al., [20]	1993	Sweden	184 Cases: 61 Con: 123	Cases: Women, aged 54–75 years, DRF 6 weeks-3 months or 11–13 months before Con: Healthy, age-matched women, no DRF	NR	Cases 6 weeks-3 months post DRF: 64 (6) Cases 11–13 months post DRF: 66 (5) Con: 64 (6) 100% women (184)	NR	N/A	Single leg balance
Sakai et al., [27]	2010	Japan	106 Cases: 54 Con: 52	Cases: Women, aged ≥ 50 years, DRF following fall from standing height, surgically treated with volar locking plate and no cast Con: Community dwelling, aged ≥ 50 years, no DRF	NR	Cases: 69.3 (9.9) Con: 67 (8.7) 100% women (106)	NR	N/A	Single leg balance eyes open
Sharabiani et al., [16]	2019	Iran	80 Cases: 40 Con: 40	Cases: 6–24 months after DRF following a fall Con: No history of DRF, age- and sex-matched	In previous 1 year Cases: 1 (1) Con: 0 (0)	Cases: 56 (4) Con: 55 (7) 83% female (66)	NR	N/A	Postural sway
**RCTs**									
Armstrong et al., [13]	1996	UK	116 Int: 57 Con: 59	Post-menopausal women, aged 45–70 years, ≤ 7 weeks after DRF, no contraindications to HRT	NR	Int: 60.5 (6.3) Con: 61.3 (5.8) 100% women (116)	HRT and calcium supplement (1000 mg/day)	Calcium supplement (1000 mg/day)	Leg extension power Postural sway
Baldursdottir et al., [33]	2020	Iceland	^II^98 Int: 47 Con: 48	2–5 months after DRF following a fall	Falls in previous 12 months Int: Median 1 (range 1–5; included fall at time of DRF) Con: Median 1 (1–6)	^#^Int: 60.8 (6.7) ^#^Con: 62.7 (7.9) 87% female (85)	Multi-sensory balance training (6 individual supervised sessions over 3 months and daily home exercise)	Wrist strengthening and coordination exercise (6 individual supervised sessions over 3 months and daily home exercise)	5 STS Sensory Organization Test
Hansson et al., [34]	2015	Sweden	85 Int: 41 Con: 44	Aged ≥ 50 years, DRF following a fall	NR	^#^Int: 73 (8) ^#^Con: 72 (10) 95% women (81)	Group-based vestibular rehabilitation x 2/week for 9 weeks	No intervention	5 STS Tandem stand Postural sway Heel-to-toe walk Figure-of-8 walk Single leg balance
Wong et al., [35]	2019	China	90 Int: NR Con: NR	Aged ≥ 60 years, 6 weeks to 3 months after DRF following a fall	NR	NR	Low magnitude high frequency vibration involving vertical synchronous vibration at 35 Hz, 0.3 g for 20 min/day x 5/week for 3 months	Continue usual lifestyle and not use a vibration machine	Balance on the Biodex Balance System
**Case series**									
Crockett et al., [30]	2019	Canada	78	Women aged ≥ 50 years, ≤ 1 week after DRF treated surgically or non-surgically	NR	^#^63 (8.4) 100% women (78)	^††^After cast removal, all participants received a standardised written home exercise programme of flexibility and strengthening exercise for the affected limb. 20 (26%) participants also reported receiving additional unspecified physiotherapy		30s STS Functional Reach Test Single leg balance
Dewan et al., [32]	2019	Canada	190	Aged 50–80 years with a DRF	NR	62.1 (7.7) 86% women (163)	NR		Knee extension strength Ankle plantarflexion strength Balance on the Biodex Balance System
Maeda et al., [29]	2021	Japan	99	Postmenopausal women, DRF following a fall treated surgically or non-surgically, independently mobile	NR	^#^70.1 (8.4) 100% women (99)	45/88 (53%) participants who completed follow-up received eldecalcitol (vitamin D3 analogue) alone. Participants also received unspecified rehabilitation	40 (48%) participants received eldecalcitol and a bone resorption inhibitor (bisphosphonate or denosumab). Participants also received unspecified rehabilitation	Knee extension strength
Mehta et al., [15]	2015	Canada	21	Aged ≥ 45 years, DRF following a fall, treated surgically or non-surgically, English primary spoken language	2/21 (9.5%) participants had ≥ 1 fall in previous 1 year	62.6 (7.6) 100% women (21)	Participants started unspecified hand therapy 44.8 (4.3) days after DRF		30s STS Hip flexion strength Hip extension strength Hip abduction strength Knee flexion strength Knee extension strength Functional Reach Test Single leg balance
Nordell et al., [31]	2003	Sweden	43	Women with a DRF following a fall	12/43 (30%) cases fell in the previous 1 year, 11/43 (26%) cases had a separate fall-related fracture in the previous 10 years	68 (8.4) 100% women (43)	NR		Single leg balance

Data are mean (standard deviation) unless otherwise stated; ^†^Falls history before DRF for cases and before enrolment for controls unless otherwise stated; ‡There were 36 cases who were divided into 18 pairs, age was averaged for both members of pairs to derive a single age for each pair; ^§^Cases were from a RCT comparing a 6-month strengthening and balance exercise programme and osteoporosis education versus osteoporosis education only but it was unclear if participants were assessed before starting theses interventions; ^¶^Participants received a variety of health interventions, so we reported those most likely to affect lower limb muscle strength and balance; ^II^Treatment allocation for 3/98 participants who did not begin allocated treatment was not reported; ^#^Data only reported for participants who completed follow-up; ^††^An unspecified number of participants were involved in an RCT that compared this standard rehabilitation programme and grip strength training of the unaffected limb versus standard rehabilitation alone; 5 STS: 5 times sit-to-stand test; 30s STS: 30 second sit-to-stand test; BBS: Berg Balance Scale; BMD: Bone mineral density; Con: Control group; DRF: Distal radius fracture; HRT: Hormone replacement therapy; Int: Intervention group; mg/day: milligrams per day; MCToSIoB: Modified Clinical Test of Sensory Integration of Balance; n: number of participants; N/A: Not applicable; NR: Not reported; SD: Standard deviation; UC: Unclear

### Lower limb muscle strength assessments in included studies

Lower limb muscle strength was assessed in 12/19 (63%) studies using 10 different methods. Knee extensor strength was most commonly assessed (6/12 (50%) studies) followed by the five times sit-to-stand test (3/12 (25%) studies) and the 30 s sit-to-stand test (3/12 (25%) studies). More broadly, assessments could be categorised into those that assessed strength during an isolated joint movement (used in 6/12 (50%) studies; hip flexion, hip extension, hip abduction, knee extension, knee flexion, ankle dorsiflexion, and ankle plantarflexion) or during a functional movement (used in 7/12 (58%) studies; five times sit-to-stand test, 30 s sit-to-stand test, leg extension power). Reported durations from distal radius fracture to lower limb muscle strength assessment ranged from 1 to 2 weeks to 24 months.

### Balance assessments in included studies

Balance was assessed in 18/19 (95%) studies using 14 different methods. Single leg balance was most commonly assessed (6/18 (33%) studies), followed by postural sway (3/18 (17%) studies) and the functional reach test (3/18 (17%) studies). Five out of 14 (36%) balance assessment methods require specialist equipment not routinely available in clinical settings (used in 8/18 studies (41%): postural sway, Sensory Organisation Test, dynamic postural stability, Modified Clinical Test of Sensory Integration of Balance, balance on Biodex Balance System). Reported durations from distal radius fracture to balance assessment ranged from 1 to 2 weeks to 24 months.

### Quality assessment

Full methodological quality assessments of included studies are presented in Additional file 4. Two of 10 (20%) case-control studies [26, 27] scored the maximum of eight stars on the modified Newcastle-Ottawa scale, two studies (20%) [14, 28] scored seven stars, three studies (30%) [19–21] scored six stars, and one study (10%) scored five stars [16], four stars [17] and three stars [18]. Seven case-control studies lost a star for ‘representativeness of cases’ [14, 16–21]. Two of the five (40%) case series [29, 30] scored the maximum five stars on the modified Newcastle-Ottawa scale, two case series (40%) [15, 31] scored four stars, and one (20%) [32] scored three stars. Two of the four (50%) RCTs [33, 34] were judged at high risk of bias in ≥ 2 domains of the Cochrane risk-of-bias tool.

### Lower limb muscle strength results

*In adults aged ≥ 50 years with a distal radius fracture compared to age- and sex/gender-matched controls*

Five included case-control studies compared lower limb muscle strength between adults aged ≥ 50 years with a distal radius fracture with age- and sex/gender-matched controls [17, 18, 21, 26, 28]. Detailed results are presented in Table 2.

**Table 2:** Lower limb muscle strength in older adults with a distal radius fracture compared with controls

Study	Number of participants analysed	Falls history	Strength assessment	^†^Device used; ^†^contraction type; units	^‡^Timepoint	Results summary		
						Cases	Controls	Between group mean difference (95% CI)
**Control participants had no recent falls**								
Cho et al., [26]	Cases: 40 Con: 40	Cases: NR Con: 0 in previous 2 years	5 STS	s	6 months	11.2 (1.9)	10.4 (1.5)	NR, p = 0.018
O’Reilly et al., [28]	Cases: 41 Con: 41	In previous 1 year Cases: 15/41 (37%) had > 1 fall Con: 0	Knee extension	HHD; isometric; UC	12 months	R: 4.91 (1.04) L: 4.82 (1.14)	R: 4.76 (0.66) L: 4.73 (0.91)	NR, R: p = 0.42 NR, L: p = 0.71
			Knee flexion			R: 3.81 (1.52) L: 3.84 (1.62)	R: 4.78 (1.60) L: 4.86 (1.62)	NR, R: p = 0.01 NR, L: p = 0.02
			Ankle dorsiflexion			R: 5.11 (0.86) L: 5.08 (0.98)	R: 5.21 (0.57) L: 5.26 (0.44)	NR, R: p = 0.52 NR, L: p = 0.32
**Cases and controls had recent falls**								
Edwards et al., [18]	Cases: 26 Con: 24	All participants averaged 1 fall in previous 12 months	Knee extension	Spring gauge; isometric; kg	6–24 months	Dominant leg: 21.5 (6.5)	Dominant leg: 22.8 (7.0)	NR (-2.5 to 5.3), p = 0.579
**Falls history NR**								
Crockett et al., [17]	^§^Cases: 32 ^§^Con: 42	NR	30s STS	Reps	6–24 months	^§^11.9 (3.5)	^§^14.7 (4.1)	-2.8 (-4.6 to -1), p = 0.003
Hakestad et al., [21]	Cases: 36 (divided into 18 pairs) Con:18	NR	Knee extensor peak torque at 60 °/sec, and total work at 180 °/sec	ID; conc; Nm (peak torque) and joules (total work)	1.3 (0.6) years	Peak torque: R: 102.4 L: 96.4	Peak torque: R: 113.2 L: 115	Peak torque: R: -10.8 (-26.9 to 5.4), p = 0.178 L: -18.6 (-34.6 to -2.6), p = 0.025
						Total work: R: 1276.8 L: 1194.8	Total work: R: 1536.7 L: 1480.4	Total work: R: -259.9 (-464.9 to -54.9), p = 0.016 L: -285.6 (-492.7 to -78.5), p = 0.010

Data are mean (standard deviation) unless otherwise stated; ^†^Only applies to instrumented measurements; ^‡^Time after distal radius fracture (does not apply to control participants); ^§^Different values reported in Crockett et al., [17] and multiple report Crockett et al., [56], so values from Crockett et al., [56] were used as this reported provided the between group mean difference (95% CI); °/sec: Degrees per second; 5 STS: 5 times sit-to-stand test; 30s STS: 30 second sit-to-stand test; CI: Confidence interval; Conc: Concentric; Con: Control group; HHD: Hand-held dynamometer; ID: Isokinetic dynamometer; kg: Kilograms; L: Left leg; N: Newtons; Nm: Newton meters; NR: Not reported; R: Right leg; Reps: Repetitions; s: seconds; UC: Unclear

Control participants had no recent falls in two case-control studies that assessed lower limb muscle strength [26, 28]. Cho et al., [26] found women six months after distal radius fracture performed worse on the five times sit-to-stand test than age- and gender-matched controls with a unilateral upper-limb condition (cases: mean 11.2 (Standard Deviation (SD) 1.9) seconds, controls: 10.4 (1.5) seconds; p = 0.018). O’Reilly et al., [28] found older adults 12 months after distal radius fracture had worse knee flexion strength than healthy age- and gender-matched controls (right leg cases: 3.81 (1.52), controls: 4.78 (1.6), p = 0.01; left leg cases: 3.84 (1.62), controls: 4.86 (1.62), p = 0.02), but there was no difference in knee extension or ankle dorsiflexion strength between groups.

In Edwards et al., there was no difference in knee extension strength of the dominant leg between women 6–24 months after distal radius fracture [18] and age- and gender matched controls who both averaged one fall in the previous 12 months.

Participants’ falls history was not reported in two case-control studies that assessed lower limb muscle strength [17, 21]. Crockett et al., [17] found women 6–24 months after distal radius fracture performed worse on the 30 s sit-to-stand test than age-matched women with no distal radius fracture (mean difference: -2.8 (95% confidence interval (CI) -4.6 to -1) repetitions, p = 0.003), and Hakestad et al., [21] found post-menopausal osteopenic women mean 1.3 (SD 0.6) years after distal radius fracture had worse knee extensor peak torque in the left leg (mean difference − 18.6 (95% CI -34.6 to -2.6) Newton metre (Nm), p = 0.025) and worse total work in both legs (mean difference right leg: -259.9 (95% CI -464.9 to -54.9) joules, p = 0.016; left leg: -285.6 (95% CI -492.7 to -78.5), p = 0.01) compared to healthy age-matched women.

### In adults aged ≥ 50 years with a distal radius fracture in all included studies

Detailed results from the 12 included studies that assessed lower limb muscle strength are presented in Table 3. Studies are listed in order of shortest duration after distal radius fracture to lower limb muscle strength assessment.

**Table 3:** Lower limb muscle strength in adults aged ≥ 50 years with a distal radius fracture

Study	Study design	Number of participants analysed	Strength assessment	^†^Device used; ^†^contraction type; units	^‡^Timepoint	Results	Change from baseline
Dewan et al., [32]	Case series	50–64 years: 121 65–80 years: 69	Knee extension right leg	ID; isometric; Nm	1–2 weeks	50–64 years: 90.6 (39.9) 65–80 years: 74.9 (29.7)	N/A
			Ankle plantarflexion right leg			50–64 years: 67.8 (37.5) 65–80 years: 57.06 (33.2)	
Crockett et al., [30]	Case series	63	30s STS	Reps	3 weeks	13.6 (4.7)	N/A
					12 weeks	14.5 (4.5)	NR
					26 weeks	15.1 (4.4)	NR
					52 weeks	15.1 (4.6)	NR
Mehta et al., [15]	Case series	21	30s STS	Reps	7 weeks	12.4 (2.9)	N/A
			Hip flexion	HHD; isometric; kg		Dominant leg: 12.5 (1.41) Non-dominant leg: 11.6 (1.32)	
			Hip extension			Dominant leg: 10.2 (2.49) Non-dominant leg: 10 (2.63)	
			Hip abduction			Dominant leg: 14.8 (2) Non-dominant leg: 14.5 (1.81)	
			Knee extension			Dominant leg: 24.1 (1.54) Non-dominant leg: 23.4 (2.72)	
			Knee flexion			Dominant leg: 15.2 (2) Non-dominant leg: 14.1 (1.68)	
Maeda et al., [29]	Case series	85	^§^Knee extension	Knee extensor strength training equipment with measurement device; isotonic; Nm	6–8 weeks	^¶^290	N/A
					58–60 weeks	296	NR, p < 0.05
Baldursdottir et al., [33]	RCT	Int: 38 Con: 42	5 STS	s	Baseline (2–5 months after DRF)	Int: ^¶^11.7 (2.61) Con: ^¶^11.4 (2.41)	N/A
		Int: 38 Con: 42			13 weeks after baseline	Int: NR Con: NR	Int: -1.5 (95% CI -1.964 to -0.996), p < 0.001 Con: -1.0 (95% CI -1.537 to -0.444), p < 0.01
Armstrong et al., [13]	RCT	Int: 54 Con: 54	^§^Leg extensor power	Leg extensor rig; conc; N	Baseline (≤ 3 months after DRF)	Int: ^¶^138 (37) Con: ^¶^147 (35)	N/A
		Int: 54 Con: 54			24 weeks after baseline	Int: 137 (42) Con: 151 (34)	Int: -0.76 (17) Con: 6.1 (19)
Cho et al., [26]	Case-control	Cases: 40	5 STS	s	6 months	11.2 (1.9)	N/A
Crockett et al., [17]	Case-control	Cases: 32	30s STS	Reps	6–24 months	11.9 (3.5)	N/A
Edwards et al., [18]	Case-control	Cases: 26	Knee extension dominant leg	Spring gauge; isometric; kg	6–24 months	21.5 (6.5)	N/A
O’Reilly et al., [28]	Case-control	Cases: 41	Knee extension	HHD; isometric; UC	12 months	R: 4.91 (1.04) L: 4.82 (1.14)	N/A
			Knee flexion			R: 3.81 (1.52) L: 3.84 (1.62)	
			Ankle dorsiflexion			R: 5.11 (0.86) L: 5.08 (0.98)	
Hakestad et al., [21]	Case-control	Cases: 36 (divided into 18 pairs)	Knee extensor peak torque at 60 °/sec and total work at 180 °/sec	ID; conc; Nm (peak torque) and joules (total work)	1.3 (0.6) years	Peak torque: R: 102.4 L: 96.4	N/A
						Total work: R: 1276.8 L: 1194.8	
Hansson et al., [34]	RCT	Int: 27 Con: 41	5 STS	s	^II^Baseline	All: ^¶^10.3 (2.7) Int: ^¶^10.7 (2.8) Con: ^¶^10.2 (2.6)	N/A
		Int: 27 Con: 41			3 months after baseline	Int: 10.2 Con: 9.3	NR

Data are mean (standard deviation) unless otherwise stated; ^†^Only applies to instrumented measurements; ^‡^Time after distal radius fracture unless otherwise stated; ^§^Leg assessed not specified; ^¶^Only data for participants who completed follow-up assessment for this outcome reported; ^II^Time after distal radius fracture not reported; °/sec: Degrees per second; 5 STS: 5 times sit-to-stand test; 30s STS: 30 second sit-to-stand test; CI: Confidence interval; Conc: Concentric; Con: Control group; DRF: Distal radius fracture; HHD: Hand-held dynamometer; ID: Isokinetic dynamometer; Int: Intervention group; kg: Kilograms; L: Left leg; N/A: Not applicable; N: Newtons; Nm: Newton meters; NR: Not reported; R: Right leg; RCT: Randomised controlled trial; s: Seconds; UC: Unclear

Five studies evaluated lower limb muscle strength at multiple time points [13, 29, 30, 33, 34], with four studies [29, 30, 33, 34] reporting strength progressively improved from initial assessment after distal radius fracture to extended follow-up.

Additional file 5 presents lower limb muscle strength outcomes by assessment method. Methods of assessing and reporting knee extension and flexion strength were highly variable limiting comparisons between studies. In participants with a distal radius fracture, mean five times sit-to-stand performance ranged from 9.3 to 11.7 s and mean 30 s sit-to-stand performance ranged from 11.9 to 15.1 repetitions.

### Balance results

*In adults aged ≥ 50 years with a distal radius fracture compared to age- and sex/gender-matched controls*

Ten included case-control studies compared balance between adults aged ≥ 50 years with a distal radius fracture with age- and sex/gender-matched controls. Detailed results are presented in Table 4.

**Table 4:** Balance in older adults with a distal radius fracture compared to controls

Study	Number of participants analysed	Falls history	Balance assessment	^†^Device used; units	Results summary			
					^‡^Timepoint	Cases	Controls	Between group mean difference (95% CI)
**Control participants had no recent falls**								
Cho et al., [26]	Cases: 40 Con: 40	Cases: NR Con: 0 in previous 2 years	^§^Balance component of Short Physical Performance Battery	^¶^Points: 0–4	6 months	3.7 (0.5)	3.7 (0.6)	NR, p = 0.68
^II^Sharabiani et al., [16]	Cases: 40 Con: 40	In previous 1 year Cases: 1 (1) Con: 0 (0)	Postural sway, standing barefoot, both feet on force plate, eyes open	Kistler force plate; cm/s (mean velocity)	6–24 months	4.1 (0.8)	4.1 (0.4)	0.07 (-0.07 to 0.22), p = 0.54
			Postural sway, standing barefoot, both feet on foam on force plate, eyes open			5.4 (0.8)	4.8 (0.5)	0.59 (0.44 to 0.73) p < 0.001
O’Reilly et al., [28]	Cases: 41 Con: 41	In previous 1 year Cases: 15/41 (37%) had > 1 fall Con: 0	MCToSIoB	UC; ^¶^score	12 months	316.2 (63.32)	353.73 (17.72)	NR, p = 0.001
**Cases and control participants had recent falls**								
Edwards et al., [18]	Cases: 26 Con: 24	All participants averaged 1 fall in previous 12 months	BBS	^¶^Score: 0–56	6–24 months	51.8 (3.9)	54.0 (2.0)	NR (0.145 to 18.2), p = 0.10
Louer et al., [19]	Cases: 23 Con: 23	In previous 1 year Cases: median 1 (SD 2.2) Con: median 0 (SD 1.3)	Dynamic postural stability	PROPRIO 5000; ^#^Dynamic motion analysis score (range: 0-1440)	6–24 months	933 (172)	790 (175)	NR, p = 0.008
				PROPRIO 5000; s (duration maintaining balance)		50.1 (17.4)	63.8 (15.9)	NR, p = < 0.01
**Falls history NR**								
Fujita et al., [14]	Cases: 128 Con: 128	NR	Functional Reach Test	cm	2 weeks after DRF surgery	Data are median (95% CI) < 55 years: 30.5 (27.7 to 33.3) 55–64 years: 30.3 (28.1 to 32.5) 65–74 years: 31.0 (29.4 to 32.5) > 74 years: 26.6 (24.5 to 28.7)	^††^Data are median (95% CI) < 55 years: 34.6 (32.7 to 36.5) 55–64 years: 30.9 (29.1 to 32.7) 65–74 years: 30.1 (28.9 to 31.3) > 74 years: 26.3 (23.3 to 29.3)	< 55 years: NR, p = 0.03 55–64 years: NR, p = 0.68 65–74 years: NR, p = 0.37 > 74 years: NR, p = 0.87
	Cases: 128				6 months after DRF surgery	< 55 years: 34.1 (32.3 to 35.9) 55–64 years: 32.4 (27.8 to 37.0) 65–74 years: 31.2 (29.4 to 32.9) > 74 years: 27.7 (25.5 to 30.0)		< 55 years: NR, p = 0.76 55–64 years: NR, p = 0.85 65–74 years: NR, p = 0.29 > 74 years: NR, p = 0.49
Ringsberg et al., [20]	Cases 1: 41 Cases 2: 20 Con: 123	NR	Single leg balance, each leg, eyes open and closed	s (max 30 per leg, max total 120)	Cases 1: 6 weeks-3 months	Cases 1: 41.2 (25.2)	58 (24.2)	NR, p < 0.001
					Cases 2: 11–13 months	Cases 2: 58.2 (26.5)		Not statistically significant, p value NR
Sakai et al., [27]	Cases: 54 Control: 52	NR	Single leg balance, dominant leg, eyes open	s (max 121)	6 months after surgery	< 15s: 44.4% ≥ 15s to ≤ 60s: 27.8% > 60s to ≤ 120s: 7.4% > 120s: 20.4%	< 15s: 13.5% ≥ 15s to ≤ 60s: 23% > 60s to ≤ 120s: 13.5% > 120s: 50%	NR
Crockett et al., [17]	Cases: 30 Con: 44	NR	BBS	^¶^Score: 0–56	6–24 months	53.9 (5.8)	55.4 (1.2)	3%, p = 0.046
	Cases: 30 Con: 44		Functional reach test component of BBS	cm		29.6 (7.7)	33.4 (5.8)	NR
	Cases: 32 Con: 45		Backwards tandem walk	Number of errors		0: n = 15 (46.9%) 1–5: n = 8 (25%) > 5: n = 6 (18.8%) No attempt: n = 3 (9.4%)	0: n = 27 (60%) 1–5: n = 11 (24.4%) > 5: n = 7 (15.6%) No attempt: n = 0 (0%)	NR
Hakestad et al., [21]	Cases: 36 (divided into 18 pairs) Con:18	NR	Four Square Step Test	^#^s	1.3 (0.6) years	9.4	7	2.4 (1.0 to 3.7), p = 0.002

Data are mean (standard deviation) unless otherwise stated; ^†^Only applies to instrumented measurements; ^‡^Time after distal radius fracture for cases unless otherwise stated; ^§^Includes tandem, semi-tandem, and feet side-by-side stands; ^¶^Higher score better; ^II^Due to the large number of postural sway outcomes reported for this study, the most relevant outcomes are included in the table, additional outcomes are presented in Additional file 6; ^#^Lower score better; ^††^Control group only assessed once; BBS: Berg Balance Scale; CI: Confidence interval; cm: centimetres; cm/s: centimetres per second; Con: Control group; DRF: Distal radius fracture; Int: Intervention group; Max: Maximum; MCToSIoB: Modified Clinical Test of Sensory Integration of Balance; n: Number of participants; N/A: Not applicable; NR: Not reported; s: Seconds; UC: Unclear

Cases had no recent falls in three case-control studies that assessed balance [16, 26, 28]. Cho et al., [26] found no difference in performance of the balance component of the short physical performance battery between women six months after distal radius fracture and age- and gender-matched controls with a unilateral upper-limb condition. Sharabiani et al., [16] found older adults 6–24 months after distal radius fracture had more postural sway than age- and sex-matched controls, but only when standing on foam (mean difference: 0.59 (95% CI 0.44 to 0.73) centimetres per second, p < 0.001). O’Reilly et al., [28] found older adults 12 months after distal radius fracture performed worse on the Modified Clinical Test of Sensory Integration of Balance than healthy age- and gender-matched controls (cases: 316.2 (63.32), controls 353.73 (17.72), p = 0.001).

Cases and control participants had recent falls in two studies that assessed balance [18, 19]. In Edwards et al., [18] there was no statistically significant difference in Berg Balance Scale performance between women 6–24 months after distal radius fracture and age- and gender-matched controls. In contrast, Louer et al., [19] found people 6–24 months after distal radius fracture performed worse on the PROPRIO 5000 than age- and sex-matched people with no distal radius fracture (dynamic motion analysis score cases: 933 (172), controls: 790 (75), p = 0.008; duration cases: 50.1 (17.4) seconds, controls: 63.8 (15.9), p = < 0.01).

Participants’ falls history was not reported in five case-control studies that assessed balance [14, 17, 20, 21, 27]. Hakestad et al., [21] found postmenopausal osteopenic women mean 1.3 (SD 0.6) years after distal radius fracture performed worse on the four-square step test than healthy age-matched women (mean difference: 2.4 (95% CI 1 to 3.7), p = 0.002). Crockett et al., [17] found post-menopausal women 6–24 months after distal radius fracture had worse Berg Balance Scale scores than age- and gender matched controls (cases: 53.9 (5.9), controls: 55.4 (1.2), p = 0.046), and also reported worse functional reach test and backwards tandem walk performance in cases, but no statistical comparison between groups for these tests were reported. Similarly, Sakai et al., [27] reported worse single leg balance performance in women six months after distal radius fracture surgery compared to age- and gender-matched controls, but no statistical comparison of results between groups was reported. In Ringsberg et al., [20] and Fujita et al., [14], only some subgroups of women with distal radius fractures performed worse than age-matched women with no distal radius fracture on the functional reach test and single leg balance test, respectively.

### Balance in adults aged ≥ 50 years with a distal radius fracture in all included studies

Detailed results from the 18 included studies that assessed balance are presented in Table 5. Seven included studies evaluated balance at multiple timepoints [13, 14, 30, 31, 33–35]. Where reported, balance performance in people with a distal radius fracture progressively improved from initial assessment to follow-up in three studies [14, 31, 33], balance performance improved on some assessments and regressed on others in two RCTs [13, 34], and in Crockett et al.,[30] functional reach test and single leg stand performance initially improved from 3 weeks after distal radius fracture until 12 weeks and 26 weeks respectively, but improvements were not maintained at 52 weeks [35].

**Table 5:** Balance in older adults with a distal radius fracture

Study	Study design	Number of participants analysed	Balance assessment	^†^Device used; units	^‡^Timepoint	Results	Change from baseline
Dewan et al., [32]	Case series	50–64 years: 121 65–80 years: 69	Balance on Biodex Balance System	Biodex Balance System; ^§^Biodex stability index	1–2 weeks	50–64 years: 2.1 (1.2) 65–80 years: 2.3 (1.1)	N/A
Fujita et al., [14]	Case series	Cases: 128	Functional Reach Test	cm	2 weeks after DRF surgery	Data are median (95% CI) < 55 years: 30.5 (27.7 to 33.3) 55–64 years: 30.3 (28.1 to 32.5) 65–74 years: 31 (29.4 to 32.5) > 74 years: 26.6 (24.5 to 28.7)	N/A
		Cases: 128			6 months after DRF surgery	< 55 years: 34.1 (32.3 to 35.9) 55–64 years: 32.4 (27.8 to 37) 65–74 years: 31.2 (29.4 to 32.9) > 74 years: 27.7 (25.5 to 30)	NR
Crockett et al., [30]	Case series	63	Functional Reach Test	cm	3 weeks	32 (5.84)	N/A
					12 weeks	34 (6.35)	NR
					26 weeks	33.3 (6.35)	
					52 weeks	31.2 (6.35)	
			Single leg balance, as long as possible, eyes open, on preferred leg		3 weeks	29.5 (19.7)	N/A
					12 weeks	30.8 (20.35)	NR
					26 weeks	34.9 (19.9)	
					52 weeks	33.4 (19.9)	
Nordell et al., [31]	Case series	43	Single leg balance, eyes open	s (max 30)	22 (14.6) days	R: 23.3 (9.1) L: 22.7 (10.5)	N/A
		43			12 months	NR	R: 1 (7.2), p = 0.3 L: 0.7 (6.8), p = 0.5
Maeda et al., [29]	Case series	21	Functional Reach Test	cm	7 weeks	37.2 (5.2)	N/A
			Single leg balance, as long as possible, eyes open	s		Dominant leg: 57.1 (29.9) Non-dominant leg: 62.5 (32.5)	
Ringsberg et al., [20]	Case-control	Cases 1: 41	Single leg balance on each leg, eyes open and closed	s (max 30 per leg, max total 120)	Cases 1: 6 weeks-3 months	Cases 1: 41.2 (25.2)	N/A
		Cases 2: 20			Cases 2: 11–13 months	Case 2: 58.2 (26.5)	
Wong et al., [35]	RCT	90 (per treatment group NR)	Overall stability index	Biodex Balance System; UC	Baseline (6 weeks–3 months after DRF)	NR	N/A
			Anteroposterior stability index				
			Medial/lateral stability index				
			Limits of stability				
			Overall stability index		3 months after baseline	Significant improvement in int compared to con, p = 0.049	NR
			Anteroposterior stability index			NR	
			Medial/lateral stability index			Significant improvement in int compared to con, p = 0.046	
			Limits of stability			Significant improvement in int compared to con, p = 0.049	
Baldursdottir et al., [33]	RCT	Int: 38 Con: 42	Sensory Organization Test	Neurocom Smart Balance Master; ^§¶^composite score of six sensory conditions	Baseline (2–5 months after DRF)	Int: ^II^74 (7.8) Con: ^II^72 (7.4)	N/A
		Int: 38 Con: 42			13 weeks after baseline	Int: NR Con: NR	Int: 4.2 (95% CI 1.495 to 6.943), p < 0.01 Con: 3.6 (95% CI 1.363 to 5.813), p < 0.01
Armstrong et al., [13]	RCT	Int: 53 Con: 54	Lateral sway, feet together, eyes closed	Wright ataxiameter; degrees	Baseline (≤ 3 months after DRF)	Int: ^II^5.58 (2.1) Con: ^II^5.3 (2.04)	N/A
			Lateral sway, feet together, eyes open			Int: ^II^4.01 (1.61) Con: ^II^3.75 (1.3)	
		Int: 53 Con: 54	Lateral sway, feet together, eyes closed		24 weeks after baseline	Int: 5.64 (1.93) Con: 4.99 (2.08)	Int: 0.027 (1.21) Con: -0.36 (1.61)
			Lateral sway, feet together, eyes open			Int: 3.82 (1.37) Con: 3.58 (0.89)	Int: -0.19 (1.23) Con: -0.21 (0.93)
Cho et al., 2014[26]	Case-control	Cases: 40	^#^Balance component of Short Physical Performance Battery	^§^Points: 0–4	6 months	3.7 (0.5)	N/A
Sakai et al., [27]	Case-control	Cases: 54	Single leg balance, dominant leg, eyes open	s (max 121)	6 months after surgery	< 15s: 44.4% ≥ 15s to ≤ 60s: 27.8% > 60s to ≤ 120s: 7.4% > 120s: 20.4%	N/A
Crockett et al., [17]	Case-control	Cases: 30	BBS	^§^Score: 0–56	6–24 months	53.9 (5.8)	N/A
		Cases: 30	Functional reach test component of BBS	cm		29.6 (7.7)	
		Cases: 32	Backwards tandem walk	Number of errors		0: n = 15 (46.9%) 1–5: n = 8 (25%) > 5: n = 6 (18.8%) No attempt: n = 3 (9.4%)	
Edwards et al., [18]	Case-control	Cases: 26	BBS	^§^Score: 0–56	6–24 months	51.8 (3.9)	N/A
Louer et al., [19]	Case-control	Cases: 23	Dynamic postural stability	PROPRIO 5000; ^††^Dynamic motion analysis score (range: 0-1440)	6–24 months	933 (172)	N/A
				PROPRIO 5000; s (duration maintaining balance)		50.1 (17.4)	NR, p = < 0.01
††††Sharabiani et al., [16]	Case-control	Cases: 40	Postural sway, standing barefoot, both feet on force plate, eyes open	Kistler force plate; cm/s (mean velocity)	6–24 months	4.1 (0.8)	N/A
			Postural sway, standing barefoot, both feet on foam on force plate, eyes open			5.4 (0.8)	
O’Reilly et al., [28]	Case-control	Cases: 41	MCToSIoB	UC; ^§^score	12 months	316.2 (63.32)	N/A
Hakestad et al., [21]	Case-control	Cases: 36 (divided into 18 pairs)	Four Square Step Test	^††^s	1.3 (0.6) years	9.4	N/A
Hansson et al., [34]	RCT	Int: 27 Con: 41	Tandem stand, eyes open	s (max 30)	Baseline (time after distal radius fracture not reported)	All: ^II^28.4 (5.3) Int: ^II^28.1 (5.8) Con: ^II^28.6 (5.1)	N/A
			Tandem stand, eyes closed			All: ^II^13.3 (10.7) Int: ^II^10.5 (9.6) Con: ^II^15.3 (12.0)	
			Single leg balance, eyes open, test leg NR			All: ^II^18.1 (11.1) Int: ^II^17.1 (10.6) Con: ^II^17.5 (11.5)	
			Single leg balance, eyes closed, test leg NR			All: ^II^4.1 (4.9) Int: ^II^3.2 (2.4) Con: ^II^4.8 (6)	
			Medio-lateral sway, standing, eyes open	Force plate; mm/s		All: ^II^5 (3) Int: ^II^5 (3.6) Con: ^II^5.1 (2.5)	
			Medio-lateral sway, standing, eyes closed			All: ^II^10.2 (6.6) Int: ^II^10.1 (5.8) Con: ^II^10.3 (7.1)	
			Anteroposterior sway, standing, eyes open			All: ^II^6.5 (3.2) Int: ^II^6.6 (3.4) Con: ^II^6.5 (3.1)	
			Anteroposterior sway, standing, eyes closed			All: ^II^15.9 (12.3) Int: ^II^16.6 (13.9) Con: ^II^15.3 (11.4)	
			Heel-to-toe walk along a line	Steps outside of line		All: ^II^2.3 (3.1) Int: ^II^2.4 (3.4) Con: ^II^2.9 (2.9)	
			Figure-of-8 walk along a line			All: ^II^4.2 (5.6) Int: ^II^4.2 (5.1) Con: ^II^4.1 (5.9)	
		Int: 27 Con: 41	Tandem stand, eyes open	s (max 30)	3 months	Int: 26.3 Con: 27.2	NR
			Tandem stand, eyes closed			Int: 13.2 Con: 16	
			Single leg balance, eyes open, test leg NR			Int: 18.1 Con: 18.1	
			Single leg balance, eyes closed, test leg NR			Int: 3.8 Con: 5	
			Medio-lateral sway, standing, eyes open	Force plate; mm		Int: 4.92 Con: 4.85	
			Medio-lateral sway, standing, eyes closed			Int: 10.6 Con: 9.4	
			Anteroposterior sway, standing, eyes open			Int: 6.92 Con: 6.23	
			Anteroposterior sway, standing, eyes closed			Int: 18.1 Con: 14.6	
			Heel-to-toe walk along a line	Steps outside of line		Int: 2.2 Con: 1.6	
			Figure-of-8 walk along a line			Int: 4.5 Con: 4.1	

Data are mean (standard deviation) unless otherwise stated; †Only applies to instrumented measurements; ‡Time after distal radius fracture for cases unless otherwise stated; ^§^Higher scores better; ^II^Only data for participants that completed follow-up assessment for this outcome reported; ^¶^Six sensory conditions: (1) eyes open, nothing moving; (2) eyes closed, nothing moving; (3) eyes open, walls moving; (4) eyes open, floor moving; (5) eyes closed, floor moving; (6) eyes open walls and floor moving; ^#^Includes tandem, semi-tandem, and feet side-by-side stands; ^††^Lower score better; ††††Due to the large number of postural sway outcomes reported for this study, the most relevant outcomes are included in the table, additional outcomes are presented in Additional file 6; BBS: Berg Balance Scale; CI: Confidence interval; cm: centimetres; cm/s: centimetres per second; Con: Control group; DRF: Distal radius fracture; Int: Intervention group; L: Left leg; Max: Maximum; MCToSIoB: Modified Clinical Test of Sensory Integration of Balance; mm: millimetres; mm/s: millimetres per second; n: number of participants; N/A: Not applicable; NR: Not reported; R: Right leg; RCT: Randomised controlled trial; s: Seconds; UC: Unclear

Additional file 6 presents balance outcomes by assessment method. Methods of assessing and reporting results for single leg balance, postural sway, and the Biodex Balance System were highly variable limiting comparisons of results for these tests between studies. In participants with a distal radius fracture, mean functional reach test performance ranged from 26.6 to 37.2 cm and mean Berg Balance Scale score ranged from 51.8 to 53.9.

## Discussion

We found evidence that older adults with a distal radius fracture demonstrate impaired lower limb muscle strength and balance compared to age- and sex/gender-matched controls, but findings were inconsistent across studies and/or outcome measures. Included studies varied highly in terms of study design, quality, lower limb muscle strength and balance assessment methods used, and reporting of results. The characteristics of control participants in case-control studies also varied. This heterogeneity limited synthesis of results and requires careful consideration when interpreting the current evidence for lower limb muscle strength and balance impairments in adults aged ≥ 50 years with a distal radius fracture.

In this review, included case-control studies were cross-sectional. This differs from the classic case-control design which compares previous exposures between cases and controls to determine the association between previous exposures and a condition of interest [36]. In the classic design, recommendations to reduce bias include selecting controls independent of the exposure being investigated so that control participants do not have an abnormally high or low exposure [36]. In the cross-sectional case-control studies in this review, control participants should instead be independent of the outcomes of interest, that is control participants’ characteristics should not be associated with abnormally good or bad lower limb muscle strength and/or balance. This was not always the case. Several included case-control studies selected controls with characteristics, such as recent falls or pain. Pain is associated with increased falls risk [37, 38], and impaired balance [25] and lower limb muscle strength [24] are associated with falls risk. So, controls with pain or previous falls may have impaired lower limb muscle strength and/or balance. In other studies, participants’ falls history was unreported making interpretation of the adequacy of controls difficult. This potential source of bias is not assessed in the amended Newcastle-Ottawa Scale used to appraise case-control studies in this review.

Notwithstanding this limitation, there is some evidence that older adults with a distal radius fracture demonstrate impaired lower limb muscle strength during functional tests compared to age- and sex/gender-matched controls. Whether the magnitude of between group differences are clinically relevant is uncertain. In Cho et al., [26] the between group difference in five times sit-to-stand test performance is below the reported minimum clinically important difference (MCID) for older adults with stable chronic obstructive pulmonary disease (1.7 s) [39] and vestibular dysfunction (2.3 s) [40]. Furthermore, the precision of this difference could not be assessed because the 95% confidence interval was not reported. In Crockett et al., [17] the between group difference in 30 s sit-to-stand performance exceeds the reported major clinically important improvement (2.0 to 2.6) [41] for people with hip osteoarthritis but the wide confidence interval shows this estimate is imprecise [26, 17]. Control participants’ characteristics could also have been associated with lower limb muscle strength performance. In Cho et al., [26] controls had a painful upper limb condition, and in Crockett et al., [17] control participants’ falls history was not reported. Methodological quality also varied with Cho et al., [26] and Crockett et al., [17] scoring 8/8 and 4/8 stars, respectively, on the Modified Newcastle-Ottawa Scale. These limitations reduce confidence in the finding that lower limb muscle strength assessed functionally is impaired in older adults with a distal radius fracture compared to age- and sex/gender-matched controls.

Compared to normative values in similarly aged healthy Danish (age 60–69, mean 18.57 (SD 5.94) reps) [42], Australian (age ≥ 60, mean 15.9 (SD 5.1) reps) [43], and Hong Kong women (age 65–69, mean 15.4 (SD 4.4) reps) [44], 30 s sit-to-stand test performance was worse in people with a distal radius fracture in included studies [15, 17, 30]. Five times sit-to-stand performance in people with a distal radius fracture in included studies [26, 33, 34] was also worse than normative values in similarly aged Italian women (age 60–64 to 70–74, mean 7.9 (SD 2) to 8.7 (SD2.3) seconds) [45] and older Japanese adults (age ≥ 60, mean 8.5s; 95% CI 7.93 to 9.07) [46], but comparable to cohorts of 60–69 year old UK females (median range 10.46 to 17.19 s) [47] and a meta-analysis of reference values for 60 to 69-year-olds (mean 11.4 s; 95% CI 11.4 to 11.4) [48]. Variability in published norms for the five times sit-to-stand test may reflect differences in the evaluated populations, or differences in testing procedures, a recognised problem with this test [48].

Three case-control studies assessed lower limb muscle strength during isolated joint movements [18, 21, 28]. All assessed knee extension strength with conflicting findings. In these studies, control participants either had no recent falls [28], recent falls [18], or falls history was not reported [21]. Assessment procedures and results reporting were also inconsistent. This heterogeneity limited our ability to draw inferences on the comparative knee extension strength between older adults with a distal radius fracture and age- and gender-matched controls. The comparative ankle dorsiflexion and knee flexion strength between older adults with a distal radius fracture and age- and gender-matched controls also remains uncertain because these were only assessed in one case-control study [28].

Though lower limb muscle strength assessment using isolated joint movements was common in included studies, comparison of results between included studies and with other cohorts was limited because of variability in measurement devices, assessment procedures, measurement units, and a lack of published normative values in large cohorts of healthy older adults. Until these limitations are addressed, functional tests, such as the 30 s sit-to-stand, may be preferable when assessing lower limb muscle strength in older adults with a distal radius fracture. The 30 s sit-to-stand and five times sit-to-stand tests have relatively standardised testing procedures, established normative values in large cohorts of healthy older adults, and do not require specialist equipment.

Though results and assessment methods were inconsistent across included case-control studies, there was an overall trend of impaired balance in older adults with a distal radius fractures compared to age and sex/gender-matched controls. Seven case-control studies evaluated balance using tests that do not require specialist equipment. Two of these found no difference in balance between groups [18, 26]. However, in Cho et al., [26] controls had a painful unilateral upper limb condition, and in Edwards et al., [18] controls had a similar falls history to cases. As described previously, these characteristics are associated with impaired balance, potentially explaining why these studies did not detect a between group difference in balance performance. The five other case-control studies that assessed balance without specialist equipment found cases, or sub-groups of cases, had impaired balance compared to controls [14, 20, 21, 27, 30], though the difference in Berg Balance Scale scores in Crockett et al. [30] was below the MCID values reported for older adults with other musculoskeletal conditions [49]. These five studies did not report participants’ falls history. If control participants did have recent falls, the between group differences in balance could be smaller than if control participants had no recent falls. Three case-control studies assessed balance using specialist equipment [16, 19, 28]. All found cases performed worse than age- and sex/gender-matched controls, except for Sharabiani et al., [16] which only found a difference between cases and controls when postural sway was assessed while standing on foam. Interpretation of results from case-control studies that assessed balance needs to consider variability in control participants’ characteristics; assessment methods; and the clinical relevance, magnitude, and precision (where reported) of between group differences. Nevertheless, the available evidence indicates that older adults with a distal radius fracture may have impaired balance compared to age- and sex/gender-matched controls, though confidence in this finding is low.

Single leg balance was the most common balance assessment method, but assessment procedures and/or reporting of results differed between all studies that used this assessment method. Test parameters, such as maximum test duration, gender, and age affect single leg balance scores [50] which limits comparison of results between studies and different cohorts. When included studies results are compared against normative values for adults aged ≥ 60 years without conditions that impair balance (weighted mean 26.9 s, 95% 23.6 to 30.2) [50] and single leg stand performance with eyes closed in healthy Australian women aged ≥ 60 years (mean 4.1 (SD 4.2) seconds) [43], no consistent trend was observed. Postural sway, the functional reach test, the Berg Balance Scale, and the Biodex Balance System were the next most common balance assessment methods. Compared to some cohorts of community-dwelling 70-year-olds, participants with a distal radius fracture in included studies performed marginally worse on the Berg Balance Scale [51]. The clinical relevance of this remains unclear as there is no high-quality evidence for cut-off scores that predict future falls [52]. There was a wide range of functional reach test scores across included studies which may be attributable to variability in testing procedures which can be a problem with this test [53]. However, performance in included studies typically exceeded normative values in community-dwelling older adults aged ≥ 60 years (mean 26.6 cm, 95% CI 25.14 to 28.06) [53], indicating that functional reach test performance may not detect balance impairments in older adults with a distal radius fracture if they exist.

There was a trend of improved lower limb muscle strength and balance over time in studies that assessed participants at multiple timepoints, indicating a possible decline in lower limb muscle strength and balance in older adults after distal radius fracture. Without prospective studies that assess lower limb muscle strength and balance before and after distal radius fracture, this remains uncertain.

In future, researchers should consider large-scale robust case-control studies, or prospective observational studies that evaluate lower limb muscle strength and/or balance before and after distal radius fracture, to address current limitations in the evidence base. To facilitate synthesis of results in future systematic reviews, assessments with standardised procedures and units of measurements should be used. Prioritisation of assessments that can be used in clinical environments to enable larger-scale research, without requiring specialist equipment, and where published normative values in healthy populations already exist, should be considered. For clinicians, the results suggest that older adults with distal radius fractures may have impaired lower limb muscle strength and/or balance, compared to controls. Therefore, the available evidence supports current guidelines that recommend older patients presenting with a fall-related distal radius fracture should be assessed for muscle strength and balance deficits as part of a falls-risk assessment [7].

This review has methodological limitations. Relevant studies may have been missed: only studies published since 1990 were eligible and we did not search grey literature. To minimise the risk of missing relevant studies, we screened the reference lists of included studies and relevant systematic reviews. Two reviewers did not extract data independently, but all extracted data was checked by a second reviewer against the published report(s). We used a modified version of the Newcastle-Ottawa scale to appraise case-control studies and case series. The Newcastle-Ottawa scale has been criticised for its poor reliability [54] and attribution of equal weight to individual domains [55], but we are not aware of another appraisal tool that caters for the study designs and outcomes of interest in this systematic review.

With respect to included studies, none assessed participants before and after distal radius fracture, so it cannot be ruled out that reported impairments in case-control studies existed pre-injury. Only 5% of participants with a distal radius fracture in included studies were men and most studies conducted assessments ≥ 6 months after fracture, so findings may not be generalisable to older men or those with a recent distal radius fracture. Where relevant, between group differences were often imprecise or not reported. This has added relevance as we did not conduct a meta-analysis.

Strengths of this systematic review include prospective registration of the systematic review protocol, independent screening of titles and abstracts and full-text reports by two reviewers, and reporting of our methods and search strategy so that this systematic review is reproducible.

## Conclusions

Compared to matched control participants, there is some evidence that adults aged ≥ 50 years with a distal radius fracture have impaired lower limb muscle strength and balance, but findings are inconsistent across studies and/or outcome measures. Variability in control participants’ characteristics, study design, study quality, and lower limb muscle strength and balance assessment methods, limited synthesis of results. Given the high prevalence of distal radius fractures and the increased risk of future fractures, falls, and functional decline in older adults after this injury, this remains an area of important clinical concern. Large-scale robust case-control and/or prospective observational studies are needed to address limitations in the current literature. This would inform rehabilitation requirements for these patients. To facilitate synthesis of results in future systematic reviews, future studies should consider using lower limb muscle strength and balance assessments that have standardised assessment procedures and measurement units.

### Electronic supplementary material

Below is the link to the electronic supplementary material.

Additional file 1: Search strategy for each database

Additional file 2: Studies where it was unclear if eligibility criteria were met and reasons for inclusion

Additional file 3: Instrumented balance assessment procedures and scoring methods

Additional file 4: Full methodological quality assessments of included studies

Additional file 5: Lower limb muscle strength in adults aged ≥ 50 years with a distal radius fracture by assessment method

Additional file 6: Balance in adults aged ≥ 50 years with a distal radius fracture by assessment method

## Data Availability

The datasets used and/or analysed during the current study are available from the corresponding author on reasonable request.

## Abbreviations

CI: Confidence interval
MCID: Minimum clinically important difference
Nm: Newton metre
PROSPERO: international prospective register of systematic reviews
PRISMA: Preferred reporting items for systematic review and meta-analyses
RCT: Randomised controlled trial
SD: Standard Deviation
