# Supplementary material for: Lower limb muscle strength and balance in older adults with a distal radius fracture: a systematic review
Source: BMC Musculoskelet Disord. 2023 Sep 18;24:741. doi: 10.1186/s12891-023-06711-4 (PMC10506229; doi:10.1186/s12891-023-06711-4)
Supplement: Supplementary file 1 — Additional file 1: Search strategy for each database [file 12891_2023_6711_MOESM1_ESM.docx]

**ADDITIONAL FILE 1**

**Search strategy for each database**

| **Embase via Ovid (1974 to present), conducted 18 Jun 2020 and updated 25 May 2022** |
| --- |
| 1 Radius Fracture/ and distal*.ti,ab,kw. |
| 2 (((radius or radial) adj4 fracture*) and distal*).ti,ab,kw. |
| 3 Colles fracture/ |
| 4 colles*.ti,ab,kw. |
| 5 wrist fracture/ |
| 6 (fracture* adj3 wrist*).ti,ab,kw. |
| 7 1 or 2 or 3 or 4 or 5 or 6 |
| 8 exp lower limb/ |
| 9 ((lower adj limb*) or (lower adj extremit*) or (lower adj body)).ti,ab,kw. |
| 10 body equilibrium/ or exp body position/ |
| 11 (body adj5 (position* or postur* or equilibrium)).ti,ab,kw. |
| 12 (postur* or balanc*).ti,ab,kw. |
| 13 standing/ |
| 14 standing.ti,ab,kw. |
| 15 quadriceps femoris muscle/ |
| 16 hamstring muscle/ |
| 17 (Quadricep* or hamstring or adductor* or tibialis or soleus).ti,ab,kw. |
| 18 exp muscle strength/ |
| 19 exp muscle weakness/ |
| 20 (strength* adj8 musc*).ti,ab,kw. |
| 21 dynamometer/ |
| 22 dynamomet*.ti,ab,kw. |
| 23 gait/ |
| 24 (gait or sway).ti,ab,kw. |
| 25 stabilography/ |
| 26 posturograph*.ti,ab,kw. |
| 27 accelerometer/ or accelerometry/ |
| 28 acceleromet*.ti,ab,kw. |
| 29 (torque or torsion* or turning force).ti,ab,kw. |
| 30 torque/ |
| 31 isometrics/ |
| 32 muscle isotonic contraction/ |
| 33 isokinetic exercise/ |
| 34 ((isometric* or isotonic* or isokinetic) adj8 (exerc* or movement* or contraction* or train* or power or control* or force* or strength*)).ti,ab,kw. |
| 35 eccentric muscle contraction/ |
| 36 concentric muscle contraction/ |
| 37 ((eccentric or concentric) adj8 (exerci* or movement* or contraction* or train* or strength* or control* or power* or force*)).ti,ab,kw. |
| 38 functional reach.ti,ab,kw. |
| 39 or/8-38 |
| 40 7 and 39 |
| 41 limit 40 to yr="1990 -Current" |

| **MEDLINE via Ovid (1946 to present), conducted 18 Jun 2020 and updated 25 May 2022** |
| --- |
| 1 Radius Fractures/ and distal*.ti,ab,kw. |
| 2 (((radius or radial) adj4 fracture*) and distal*).ti,ab,kw. |
| 3 Colles' Fracture/ |
| 4 colles*.ti,ab,kw. |
| 5 (fracture* adj3 wrist*).ti,ab,kw. |
| 6 or/1-5 |
| 7 Lower Extremity/ |
| 8 ((lower adj limb*) or (lower adj extremit*) or (lower adj body)).ti,ab,kw. |
| 9 Postural Balance/ or posture/ |
| 10 (postur* or balanc*).ti,ab,kw. |
| 11 (body adj5 (position* or postur* or equilibrium)).ti,ab,kw. |
| 12 standing position/ |
| 13 standing.ti,ab,kw. |
| 14 Quadriceps muscle/ or Hamstring muscles/ |
| 15 (Quadricep* or hamstring or adductor* or tibialis or soleus).ti,ab. |
| 16 exp Muscle Strength/ |
| 17 exp Muscle Weakness/ |
| 18 strength*.ti,ab,kw. |
| 19 balanc*.ti,ab,kw. |
| 20 Muscle Strength Dynamometer/ |
| 21 dynamomet*.ti,ab,kw. |
| 22 Gait/ |
| 23 (gait or sway).ti,ab,kw. |
| 24 posturograph*.ti,ab,kw. |
| 25 Accelerometry/ |
| 26 acceleromet*.ti,ab,kw. |
| 27 (torque or power or force).ti,ab,kw. |
| 28 (isometric* or isotonic* or isokinetic).ti,ab,kw. |
| 29 (eccentric or concentric).ti,ab,kw. |
| 30 functional reach.ti,ab,kw. |
| 31 or/7-30 |
| 32 6 and 31 |
| 33 limit 32 to yr="1990 - 2021" |

| **CINAHL via NHS Athens (1981 to present), conducted 18 Jun 2020** | | |
| --- | --- | --- |
| 1 | "RADIUS FRACTURES"/ AND (distal).ti,ab |  |
| 2 | (((radius OR radial) ADJ4 fracture*) AND distal*).ti,ab |  |
| 3 | "WRIST FRACTURES"/ |  |
| 4 | (colles).ti,ab |  |
| 5 | (fracture* ADJ3 wrist*).ti,ab |  |
| 6 | (1 OR 2 OR 3 OR 4 OR 5) |  |
| 7 | exp "LOWER EXTREMITY"/ |  |
| 8 | ((lower ADJ limb*) OR (lower ADJ extremit*) OR (lower ADJ body)).ti,ab |  |
| 9 | "BALANCE, POSTURAL"/ |  |
| 10 | "BODY MECHANICS"/ |  |
| 11 | (body ADJ5 (position* OR postur* OR equilibrium)).ti,ab |  |
| 12 | (postur* OR balanc*).ti,ab |  |
| 13 | STANDING/ |  |
| 14 | (standing).ti,ab |  |
| 15 | "QUADRICEPS MUSCLES"/ OR "HAMSTRING MUSCLES"/ |  |
| 16 | (Quadricep* OR hamstring OR adductor* OR tibialis OR soleus).ti,ab |  |
| 17 | "MUSCLE STRENGTH"/ |  |
| 18 | "MUSCLE WEAKNESS"/ |  |
| 19 | (strength* ADJ8 musc*).ti,ab |  |
| 20 | (dynamomet*).ti,ab |  |
| 21 | exp GAIT/ |  |
| 22 | (gait OR sway).ti,ab |  |
| 23 | POSTUROGRAPHY/ |  |
| 24 | (posturograph*).ti,ab |  |
| 25 | ACCELEROMETERS/ |  |
| 26 | (acceleromet*).ti,ab |  |
| 27 | (torque OR torsion* OR "turning force").ti,ab |  |
| 28 | TORQUE/ |  |
| 29 | "ISOMETRIC CONTRACTION"/ |  |
| 30 | "ISOTONIC CONTRACTION"/ OR "CONCENTRIC CONTRACTION"/ OR "ECCENTRIC CONTRACTION"/ |  |
| 31 | "ISOTONIC EXERCISES"/ OR "ISOMETRIC EXERCISES"/ OR "ISOKINETIC EXERCISES"/ |  |
| 32 | ((isometric* OR isotonic* OR isokinetic) ADJ8 (exerc* OR movement* OR contraction* OR train* OR power OR control* OR force* OR strength*)).ti,ab |  |
| 33 | ((eccentric OR concentric) ADJ8 (exerci* OR movement* OR contraction* OR train* OR strength* OR control* OR power* OR force*)).ti,ab |  |
| 34 | ("functional reach").ti,ab |  |
| 35 | (7 OR 8 OR 9 OR 10 OR 11 OR 12 OR 13 OR 14 OR 15 OR 16 OR 17 OR 18 OR 19 OR 20 OR 21 OR 22 OR 23 OR 24 OR 25 OR 26 OR 27 OR 28 OR 29 OR 30 OR 31 OR 32 OR 33 OR 34) |  |
| 36 | (6 AND 35) [DT 1990-2020] |  |

| **CINAHL via EBSCOhost, conducted 25 May 2022 (the updated search was conducted via EBSCOhost as searching CINAHL via NHS Athens was no longer possible at the lead author’s institution at that time)** | |
| --- | --- |
| 37 | S36 AND DT 20200520-20220525 |
| 36 | S6 AND S35 |
| 35 | S7 OR S8 OR S9 OR S10 OR S11 OR S12 OR S13 OR S14 OR S15 OR S16 OR S17 OR S18 OR S19 OR S20 OR S21 OR S22 OR S23 OR S24 OR S25 OR S26 OR S27 OR S28 OR S29 OR S30 OR S31 OR S32 OR S33 OR S34 |
| 34 | TI "functional reach" OR AB "functional reach" |
| 33 | TI ((eccentric OR concentric) N8 (exerci* OR movement* OR contraction* OR train* OR strength* OR control* OR power* OR force*)) OR AB ((eccentric OR concentric) N8 (exerci* OR movement* OR contraction* OR train* OR strength* OR control* OR power* OR force*)) |
| 32 | TI ((isometric* OR isotonic* OR isokinetic) N8 (exerc* OR movement* OR contraction* OR train* OR power OR control* OR force* OR strength*)) OR AB ((isometric* OR isotonic* OR isokinetic) N8 (exerc* OR movement* OR contraction* OR train* OR power OR control* OR force* OR strength*)) |
| 31 | MH "Isotonic Exercises" OR MH "Isometric Exercises" OR MH "Isokinetic Exercises" |
| 30 | MH "Isotonic Contraction" OR MH "Concentric Contraction" OR MH "Eccentric Contraction" |
| 29 | MH "Isometric Contraction" |
| 28 | MH "Torque" |
| 27 | TI (torque OR torsion* OR "turning force")) OR AB (torque OR torsion* OR "turning force")) |
| 26 | TI acceleromet* OR AB acceleromet* |
| 25 | MH "Accelerometers" |
| 24 | TI posturograph* OR AB posturograph* |
| 23 | MH "Posturography" |
| 22 | TI (gait OR sway) OR AB (gait OR sway) |
| 21 | MH "Gait+" |
| 20 | TI dynamomet* OR AB dynamomet* |
| 19 | TI (strength* N8 musc*) OR AB (strength* N8 musc*) |
| 18 | MH "Muscle Weakness" |
| 17 | MH "Muscle Strength" |
| 16 | TI (Quadricep* OR hamstring OR adductor* OR tibialis OR soleus) OR AB (Quadricep* OR hamstring OR adductor* OR tibialis OR soleus) |
| 15 | MH "Quadriceps Muscles" OR MH "Hamstring Muscles" |
| 14 | TI standing OR AB standing |
| 13 | MH "Standing" |
| 12 | TI (postur* OR balanc*) OR AB (postur* OR balanc*) |
| 11 | TI (body N5 (position* OR postur* OR equilibrium)) OR AB (body N5 (position* OR postur* OR equilibrium)) |
| 10 | MH "Body Mechanics" |
| 9 | MH "Balance, Postural" |
| 8 | TI ("lower limb*" OR "limb* lower" OR "lower extremi*" OR "extremit* lower" OR "lower body" OR "body lower") OR AB ("lower limb*" OR "limb* lower" OR "lower extremi*" OR "extremit* lower" OR "lower body" OR "body lower") |
| 7 | MH "Lower Extremity+" |
| 6 | S1 OR S2 OR S3 OR S4 OR S5 |
| 5 | TI (fracture* N3 wrist*) OR AB (fracture* N3 wrist*) |
| 4 | TI colles OR AB colles |
| 3 | MH "Wrist Fractures" |
| 2 | TI (((radius OR radial) N4 fracture*) AND distal*) OR AB (((radius OR radial) N4 fracture*) AND distal*) |
| 1 | (MH "Radius Fractures") AND (TI distal OR AB distal) |
