# Supplementary material for: Lower limb muscle strength and balance in older adults with a distal radius fracture: a systematic review
Source: BMC Musculoskelet Disord. 2023 Sep 18;24:741. doi: 10.1186/s12891-023-06711-4 (PMC10506229; doi:10.1186/s12891-023-06711-4)
Supplement: Supplementary file 2 — Additional file 2: Studies where it was unclear if eligibility criteria were met and reasons for inclusion [file 12891_2023_6711_MOESM2_ESM.docx]

**ADDITIONAL FILE 2**

**Studies where it was unclear if eligibility criteria were met and reasons for inclusion**

| **Study** | **Reason(s) for inclusion** | |
| --- | --- | --- |
|  | **Age of participants** | **Duration since DRF to enrolment** |
| Armstrong et al., [13] | Included participants aged 45 to 70 years. Mean age in intervention 60.5 (SD 6.3 (n = 57)) years and in control was 61.3 (SD 5.8 (n = 59)) years.  Assuming age is normally distributed, 2 SDs (approximately 12 years) from an approximate mean of 60 indicates approximately 2.5% of participants were aged ≤48 years | N/A |
| Crockett et al., [17] | N/A | Cases were 6 to 24 months after distal radius fracture, but exact duration from fracture to enrolment was not reported. |
| Edwards et al., [18] | N/A | Cases had fallen in past 2 years and were a minimum of 6 months after distal radius fracture, but exact duration from fracture to enrolment was not reported |
| Fujita et al., [14] | Included participants aged >40 years. Mean age of cases was 66.9 (SD 9.3 (n = 128)) years, <13% were aged <55 years.  Assuming age is normally distributed, 2 SDs (18.6 years) from mean of 66.9 years indicates approximately 2.5% of participants were aged ≤48.3 years | N/A |
| Hakestad et al., [21] | N/A | Time since wrist fracture to inclusion for cases was mean 1.3 (SD 0.6) years |
| Louer et al., [19] | N/A | Cases were 6 to 24 months after distal radius fracture, but exact duration from fracture to enrolment was not reported. |
| Mehta et al., [15] | Included participants aged 45 years. Mean age of participants was 62.6 (SD 7.6 (n = 21)) years and median age was 62 (IQR 57.5 to 68)  Assuming age is normally distributed, 2 SDs (15.2 years) from mean of 62.6 indicates approximately 2.5% of participants were aged ≤47.4 years | N/A |
| Ringsberg et al., [20] | N/A | 20/61 participants underwent assessment 11-13 months after wrist fracture. Exact duration from wrist fracture to enrolment was not reported |
| Sharabiani et al., [16] | Age range of cases (n = 40) was 45 to 64 years. Mean age of cases was 56 (SD 4) years.  Assuming age is normally distributed, 2 SDs (8 years) from mean of 56 indicates approximately 2.5% of participants were aged ≤48 years | Participants were 6 to 24 months after wrist fracture. Exact duration from fracture to enrolment was not reported |

IQR: Interquartile range; n = Number of participants; N/A: Not applicable; SD: Standard deviation
