# Supplementary material for: Lower limb muscle strength and balance in older adults with a distal radius fracture: a systematic review
Source: BMC Musculoskelet Disord. 2023 Sep 18;24:741. doi: 10.1186/s12891-023-06711-4 (PMC10506229; doi:10.1186/s12891-023-06711-4)
Supplement: Supplementary file 3 — Additional file 3: Instrumented balance assessment procedures and scoring methods [file 12891_2023_6711_MOESM3_ESM.docx]

**ADDITIONAL FILE 3**

**Instrumented balance assessment procedures and scoring methods**

| **Study** | **Year** | **Assessment** | **Device/Test** | **Assessment procedure** | **Scoring method** |
| --- | --- | --- | --- | --- | --- |
| Armstrong et al., [13] | 1996 | Lateral sway | Wright ataxiameter | Lateral sway assessed twice with the eyes open and twice with the eyes closed. | The mean sway (degrees) of three measurements of 20 seconds with the feet together was taken |
| Baldursdottir et al., [33] | 2020 | Sensory Organization Test | Smart Balance Master | Participant stands still on the Smart Balance Master platform with harness attached in case of a fall. Participant has to try and stand upright and still as possible during six sensory conditions that involve tilting the visual surrounds or platform in an anterior to posterior direction. The six conditions are 1.) eyes open, platform not moving; 2.) eyes closed, platform not moving; 3.) eyes open; platform not moving but visual surround moving; (4) eyes open, platform moving; (5) eyes closed, platform moving; (6) eyes open, platform moving and visual surround moving. Participant performed three 20 seconds trials for each condition | A score of 0 to 100 is calculated for each condition (higher scores better). A composite score from the six conditions is calculated by:   1. Averaging scores from conditions 1 and 2 2. Add score from 1. to the scores of each trial for conditions 3 to 6 3. Divide 2. by the total number of trials |
| Dewan et al., [32] | 2019 | Balance on the Biodex Balance System | Biodex Balance System | Circular platform that can move anterior-posterior and medial-lateral at the same time. The platform’s stability can also be changed by changing the amount of resistance force applied to the platform. Assessment was conducted on level 12 (stable platform) and level 6 (moderately unstable platform) of the Biodex Balance System. Degrees of tilt are measured about each axis during tests. | Overall stability index which is a composite of anterior-posterior and medial-lateral stability (higher scores better) |
| Hansson et al., [34] | 2015 | Postural sway | Force plate | Performed with eyes open and eyes closed | Computer programme calculated mean speed of centre of pressure movements (mm/s) in medial-lateral and anterior-posterior directions for the eyes open and eyes closed conditions |
| Louer et al., [19] | 2016 | Dynamic postural stability | PROPRIO 5000 | Participant stands on motorised movable platform with feet shoulder width apart, knees slightly bent, and centre of mass over middle of the platform. Movement of participant's centre of mass is measured by an ultrasonic sensor placed in the L5/S1 region. The participant holds a 15.2cm rope in both hands (elbows flexed) to limit use of the upper limbs to maintain balance. Three two-minute trials were conducted with one minute between trials. Trials finished when; 1.) 2 minutes completed; 2.: participant's displacement >7.6cm in 0.25 seconds, 3.) participant moved > 12.7cm from starting position, 4.) participant let go of rope, 5.) participant moved their feet, 6.) participant asked to stop | Dynamic motion analysis score (lower scores better) calculated by software within device and duration (second) maintaining balance |
| O’Reilly et al., [28] | 2013 | Modified Clinical Test of Sensory Integration of Balance | N/A | Tests how vestibular, visual and somatosensory input influences standing balance under six conditions: standing on floor 1.) floor eyes open; 2.) eyes closed; 3.) wearing a dome that deprives peripheral vision and provides a sway-referenced image. Conditions 4-6.) involve repeating 1-3.) while standing on foam | Unclear but higher scores better |
| Sharabiani et al., [16] | 2019 | Postural sway | Kistler force plate | Participant asked to stand barefoot on force plate with feet together, hands by their side, and look straight forward. Postural control was measured on stable surface (standing on force plate) and unstable surface (standing on foam placed on force plate). Postural sway was measured under five different conditions: 1.) baseline, 2.) internal focus, 3.) external focus., 4.) easy cognitive task, 5.) difficult cognitive task. Further details for these conditions provided in study report.  Two trials performed for each condition each lasting 70 seconds with 60 seconds rest between trials. There were five-minute rests between each test condition. The order of test conditions was random. | Mean (cm/s), standard deviation, and path length (cm) of centre of pressure sway (mm/s) in medio-lateral and anteroposterior sway calculated by force plate for each test condition on stable and unstable surface |
| Wong et al., [35] | 2019 | Balance on the Biodex Balance System | Biodex Balance System | Unclear | Overall stability index, anteroposterior stability index, Medial/lateral stability index, and limits of stability |
