# Supplementary material for: Lower limb muscle strength and balance in older adults with a distal radius fracture: a systematic review
Source: BMC Musculoskelet Disord. 2023 Sep 18;24:741. doi: 10.1186/s12891-023-06711-4 (PMC10506229; doi:10.1186/s12891-023-06711-4)
Supplement: Supplementary file 4 — Additional file 4: Full methodological quality assessments of included studies [file 12891_2023_6711_MOESM4_ESM.docx]

**ADDITIONAL File 4**

**Individual domains of the Cochrane risk-of-bias tool for included randomised controlled trials (n = 4)**

| **Study** | Random sequence generation  (selection bias) | Allocation concealment  (selection bias) | Blinding of participants and personnel (performance bias) | Blinding of outcome assessment  (detection bias) | Incomplete outcome data  (attrition bias) | Selective reporting  (reporting bias) | Other bias |
| --- | --- | --- | --- | --- | --- | --- | --- |
| Armstrong et al., [13] |  |  |  |  |  |  |  |
| Baldursdottir et al., [33] |  |  |  |  |  |  |  |
| Hansson et al., [34] |  |  |  |  |  |  |  |
| Wong et al., [35] |  |  |  |  |  |  |  |

Low risk of bias Unclear risk of bias High risk of bias

**Individual domains of the Modified Newcastle-Ottawa Scale for included case-control studies (n = 10)**

|  | Domain 1: Selection | | | | Domain 2: Comparability | Domain 3: Outcome | |  |
| --- | --- | --- | --- | --- | --- | --- | --- | --- |
|  | Is the case definition adequate?  (Max: 🞷) | Representativeness of the cases  (Max: 🞷) | Selection of controls  (Max: 🞷) | Definition of controls  (Max: 🞷) | Comparability of cases and controls on the basis of design or analysis  (Max: 🞷🞷) | Ascertainment of exposure  (Max: 🞷) | Non-Response rate  (Max: 🞷) | Total score  (out of 8 stars) |
| Cho et al., [26] | 🞷 | 🞷 | 🞷 | 🞷 | 🞷 🞷 | 🞷 | 🞷 | 8 |
| Crockett et al., [17] | - | - | 🞷 | - | 🞷 🞷 | - | 🞷 | 4 |
| Edwards et al., [18] | - | - | - | - | 🞷 🞷 | - | 🞷 | 3 |
| Fujita et al., [14] | 🞷 | - | 🞷 | 🞷 | 🞷 🞷 | 🞷 | 🞷 | 7 |
| Hakestad et al., [21] | 🞷 | - | - | 🞷 | 🞷 🞷 | 🞷 | 🞷 | 6 |
| Louer et al., [19] | 🞷 | - | 🞷 | 🞷 | 🞷 🞷 | 🞷 | - | 6 |
| O’Reilly et al., [28] | 🞷 | 🞷 | 🞷 | 🞷 | 🞷 🞷 | 🞷 | - | 7 |
| Ringsberg et al., [20] | 🞷 | - | - | 🞷 | 🞷 🞷 | 🞷 | 🞷 | 6 |
| Sakai et al., [27] | 🞷 | 🞷 | 🞷 | 🞷 | 🞷 🞷 | 🞷 | 🞷 | 8 |
| Sharabiani et al., [16] | 🞷 | - | 🞷 | 🞷 | 🞷 🞷 | - | - | 5 |

**Individual domains of the Modified Newcastle-Ottawa Scale for included case series (n = 5)**

|  | Domain 1: Selection | | Domain 2: Outcome | | |  |
| --- | --- | --- | --- | --- | --- | --- |
|  | Representativeness of the exposed cohort  (Max: 🞷) | Ascertainment of exposure  (Max: 🞷) | Assessment of outcome  (Max: 🞷) | Was follow-up long enough for outcomes to occur  (Max: 🞷) | Adequacy of follow up of cohorts  (Max: 🞷) | Total score  (out of 5 stars) |
| Crockett et al., [30] | 🞷 | 🞷 | 🞷 | 🞷 | 🞷 | 5 |
| Dewan et al., [32] | 🞷 | - | 🞷 | - | 🞷 | 3 |
| Maeda et al., [29] | 🞷 | 🞷 | 🞷 | 🞷 | 🞷 | 5 |
| Mehta et al., [15] | 🞷 | 🞷 | 🞷 | - | 🞷 | 4 |
| Nordell et al., [31] | 🞷 | 🞷 | 🞷 | 🞷 | - | 4 |
