# Supplementary material for: Lower limb muscle strength and balance in older adults with a distal radius fracture: a systematic review
Source: BMC Musculoskelet Disord. 2023 Sep 18;24:741. doi: 10.1186/s12891-023-06711-4 (PMC10506229; doi:10.1186/s12891-023-06711-4)
Supplement: Supplementary file 5 — Additional file 5: Lower limb muscle strength in adults aged ≥ 50 years with a distal radius fracture by assessment method [file 12891_2023_6711_MOESM5_ESM.docx]

**ADDITIONAL FILE 5**

**Lower limb muscle strength in adults aged ≥50 years with a distal radius fracture by assessment method**

| **Study** | **Study design** | **Number of participants analysed** | **^†^Device used; ^†^contraction type; units** | **^‡^Timepoint** | **Results** | **Change from baseline** |
| --- | --- | --- | --- | --- | --- | --- |
| **Knee extension strength** | | | | | | |
| Dewan et al., [32] | Case series | 50-64 years: 121  65-80 years: 69 | ID; isometric; Nm | 1-2 weeks | Age 50-64 R: 90.6 (39.9)  Age 65-80 R: 74.9 (29.7) | N/A |
| Mehta et al., [15] | Case series | 21 | HHD; isometric; kg | 7 weeks | Dominant leg: 24.1 (1.54)  Non-dominant leg: 23.4 (2.72) | N/A |
| Maeda et al., [29] | Case series | ^§^85 | Knee extensor strength training equipment with measurement device; isotonic; Nm | 6-8 weeks | ^¶^290 | N/A |
|  |  |  |  | 58-60 weeks | 296 | NR, p <0.05 |
| Edwards et al., [18] | Case-control | Cases: 26  Con: 24 | Spring gauge; isometric; kg | 6-24 months | Dominant leg: 21.5 (6.5) | N/A |
| O’Reilly et al., [28] | Case-control | Cases: 41  Con: 41 | HHD; isometric; UC | 12 months | R: 4.91 (1.04)  L: 4.82 (1.14) | N/A |
| Hakestad et al., [21] | Case-control | Cases: 36 (divided into 18 pairs)  Con:18 | ID; conc; Nm (peak torque at 60°/sec) and joules total work at 180°/sec) | 1.3 (0.6) years | Peak torque:  R: 102.4  L: 96.4 | N/A |
|  |  |  |  |  | Total work:  R: 1276.8  L: 1194.8 |  |
| **5 STS** | | | | | | |
| Baldursdottir et al., [33] | RCT | Int: 38  Con: 42 | s | Baseline (2-5 months after DRF) | Int: ^¶^11.7 (2.61)  Con: ^¶^11.4 (2.41) | N/A |
|  |  | Int: 38  Con: 42 |  | 13 weeks after baseline | Int: NR  Con: NR | Int: -1.5 (95% CI -1.964 to -0.996), p < 0.001  Con: -1.0 (95% CI -1.537 to -0.444), p < 0.01 |
| Hansson et al., [34] | RCT | Int: 27  Con: 41 | s | ^‖^Baseline | All: ^¶^10.3 (2.7)  Int: ^¶^10.7 (2.8)  Con: ^¶^10.2 (2.6) | N/A |
|  |  | Int: 27  Con: 41 |  | 3 months after baseline | Int: 10.2  Con: 9.3 | NR |
| Cho et al., [26] | Case-control | Cases: 40  Con: 40 | s | 6 months | 11.2 (1.9) | N/A |
| **30s STS** | | | | | | |
| Mehta et al., [15] | Case series | 21 | Reps | 7 weeks | 12.4 (2.9) | N/A |
| Crockett et al., [30] | Case series | 63 | Reps | 3 weeks | 13.6 (4.7) | N/A |
|  |  |  |  | 12 weeks | 14.5 (4.5) | NR |
|  |  |  |  | 26 weeks | 15.1 (4.4) | NR |
|  |  |  |  | 52 weeks | 15.1 (4.6) | NR |
| Crockett et al., [17] | Case-control | Cases: 32  Con: 42 | Reps | 6-24 months | 11.9 (3.5) | N/A |
| **Knee flexion strength** | | | | | | |
| Mehta et al., [15] | Case series | 21 | HHD; isometric; kg | 7 weeks | Dominant leg: 15.2 (2)  Non-dominant leg: 14.1 (1.68) | N/A |
| O’Reilly et al., [28] | Case-control | Cases: 41  Con: 41 | HHD; isometric; UC | 12 months | R: 3.81 (1.52)  L: 3.84 (1.62) | N/A |

Data are mean (standard deviation) unless otherwise stated; ^†^Only applies to instrumented measurements; ^‡^Time after distal radius fracture unless otherwise stated; ^§^Assessed leg not specified; ^¶^Only data for participants that completed follow-up reported; ^‖^Time after distal radius fracture not reported; °/sec: Degrees per second; 5 STS: 5 times sit-to-stand test; 30s STS: 30 second sit-to-stand test CI: Confidence interval; Con: Control group; Conc: Concentric; DRF: Distal radius fracture; HHD: Hand-held dynamometer; ID: Isokinetic dynamometer; Int: Intervention group; kg: Kilograms; L: Left leg; N/A: Not applicable; Nm: Newton meters; NR: Not reported; R: Right leg; RCT: Randomised controlled trial; Reps: Repetitions; s: seconds; UC: Unclear
