# Supplementary material for: Lower limb muscle strength and balance in older adults with a distal radius fracture: a systematic review
Source: BMC Musculoskelet Disord. 2023 Sep 18;24:741. doi: 10.1186/s12891-023-06711-4 (PMC10506229; doi:10.1186/s12891-023-06711-4)
Supplement: Supplementary file 6 — Additional file 6: Balance in adults aged ≥ 50 years with a distal radius fracture by assessment method [file 12891_2023_6711_MOESM6_ESM.docx]

**ADDITIONAL FILE 6**

**Balance in adults aged ≥50 years with a distal radius fracture by assessment method**

| **Study** | **Study design** | **Number of participants analysed** | **Balance assessment** | **^†^Device used; units** | **^‡^Timepoint** | **Results** | **Change from baseline** |
| --- | --- | --- | --- | --- | --- | --- | --- |
| **Single leg balance** | | | | | | | |
| Hansson et al., [34] | RCT | Int: 27  Con: 41 | Eyes open, test leg NR | s (max 30) | ^§^Baseline | All: ^¶^18.1 (11.1)  Int: ^¶^17.1 (10.6)  Con: ^¶^17.5 (11.5) | N/A |
|  |  |  | Eyes closed, test leg NR |  |  | All: ^¶^4.1 (4.9)  Int: ^¶^3.2 (2.4)  Con: ^¶^4.8 (6) |  |
|  |  | Int: 27  Con: 41 | Eyes open, test leg NR |  | 3 months | Int: 18.1  Con: 18.1 | NR |
|  |  |  | Eyes closed, test leg NR |  |  | Int: 3.8  Con: 5 |  |
| Sakai et al., [27] | Case-control | Cases: 54 | Eyes open, dominant leg | s (max 121) | 6 months after surgery | <15s: 44.4%  ≥15s to ≤60s: 27.8%  >60s to ≤120s: 7.4%  >120s: 20.4% | N/A |
| Nordell et al., [31] | Case series | 43 | Eyes open | s (max 30) | 22 (14.6) days | R: 23.3 (9.1)  L: 22.7 (10.5) | N/A |
|  |  | 43 |  |  | 12 months | NR | R: 1 (7.2), p = 0.3  L: 0.7 (6.8), p = 0.5 |
| Crockett et al., [30] | Case series | 63 | As long as possible, eyes open, on participants’ preferred leg | s | 3 weeks | 29.5 (19.7) | N/A |
|  |  |  |  |  | 12 weeks | 30.8 (20.35) | NR |
|  |  |  |  |  | 26 weeks | 34.9 (19.9) | NR |
|  |  |  |  |  | 52 weeks | 33.4 (19.9) | NR |
| Ringsberg et al., [20] | Case-control | Cases 1: 41  Cases 2: 20 | Each leg, eyes open and closed | s (max 30 per leg, max total 120) | Cases 1: 6 weeks-3 months | Cases 1: 41.2 (25.2) | N/A |
|  |  |  |  |  | Cases 2: 11-13 months | Cases 2: 58.2 (26.5) |  |
| Mehta et al., [15] | Case series | 21 | As long as possible, eyes open | s | 7 weeks | Dominant leg: 57.1 (29.9)  Non-dominant leg: 62.5 (32.5) | N/A |
| **Postural sway** | | | | | | | |
| Armstrong et al., [13] | RCT | Int: 53  Con: 54 | Lateral sway, feet together, eyes closed | Wright ataxiameter; degrees | Baseline (≤3 months after DRF) | Int: ^¶^5.58 (2.1)  Con: ^¶^5.3 (2.04) | N/A |
|  |  |  | Lateral sway, feet together, eyes open |  |  | Int: ^¶^4.01 (1.61)  Con: ^¶^3.75 (1.3) | N/A |
|  |  | Int: 53  Con: 54 | Lateral sway, feet together, eyes closed |  | 24 weeks after baseline | Int: 5.64 (1.93)  Con: 4.99 (2.08) | Int: 0.027 (1.21)  Con: -0.36 (1.61) |
|  |  |  | Lateral sway, feet together, eyes open |  |  | Int: 3.82 (1.37)  Con: 3.58 (0.89) | Int: -0.19 (1.23)  Con: -0.21 (0.93) |
| Hansson et al., [34] | RCT | Int: 27  Con: 41 | Medio-lateral sway, standing, eyes open | Force plate; mm | Baseline | All: ^¶^5 (3)  Int: ^¶^5 (3.6)  Con: ^¶^5.1 (2.5) | N/A |
|  |  |  | Medio-lateral sway, standing, eyes closed |  |  | All: ^¶^10.2 (6.6)  Int: ^¶^10.1 (5.8)  Con: ^¶^10.3 (7.1) |  |
|  |  |  | Anteroposterior sway, standing, eyes open |  |  | All: ^¶^6.5 (3.2)  Int: ^¶^6.6 (3.4)  Con: ^¶^6.5 (3.1) |  |
|  |  |  | Anteroposterior sway, standing, eyes open |  |  | All: ^¶^15.9 (12.3)  Int: ^¶^16.6 (13.9)  Con: ^¶^15.3 (11.4) |  |
|  |  | Int: 27  Con: 41 | Medio-lateral sway, standing, eyes open |  | 3 months | Int: 4.92  Con: 4.85 | NR |
|  |  |  | Medio-lateral sway, standing, eyes closed |  |  | Int: 10.6  Con: 9.4 |  |
|  |  |  | Anteroposterior sway, standing, eyes open |  |  | Int: 6.92  Con: 6.23 |  |
|  |  |  | Anteroposterior sway, standing, eyes closed |  |  | Int: 18.1  Con: 14.6 |  |
| Sharabiani et al., [16] | Case-control | Cases: 40 | Postural sway, standing barefoot, both feet on force plate, eyes open | Kistler force plate; cm/s (mean velocity) | 6-24 months | 4.1 (0.8) | N/A |
|  |  |  | Postural sway, standing barefoot, both feet on foam on force plate, eyes open |  |  | 5.4 (0.8) |  |
|  |  |  | Anteroposterior sway, standing barefoot, both feet on force plate, eyes open | Kistler force plate; cm/s (SD of velocity) |  | 3.7 (0.7) |  |
|  |  |  | Anteroposterior sway, standing barefoot, both feet on foam on force plate, eyes open |  |  | 4.7 (0.7) |  |
|  |  |  | Mediolateral sway, standing barefoot, both feet on force plate, eyes open | Kistler force plate; cm/s (SD of velocity) |  | 3 (0.5) |  |
|  |  |  | Mediolateral sway, standing barefoot both feet on foam on force plate, eyes open |  |  | 4 (0.9) |  |
|  |  |  | Postural sway, standing barefoot, both feet on force plate, eyes open | Kistler force plate; cm (path length) |  | 289 (54) |  |
|  |  |  | Postural sway, standing barefoot, both feet on foam on force plate, eyes open |  |  | 377 (59) |  |
| **Functional reach test** | | | | | | | |
| Fujita et al., [14] | Case-control | Cases: 128 | Functional reach test | cm | 2 weeks after DRF surgery | Data are median (95% CI)  <55 years: 30.5 (27.7 to 33.3)  55-64 years: 30.3 (28.1 to 32.5)  65-74 years: 31 (29.4 to 32.5)  >74 years: 26.6 (24.5 to 28.7) | N/A |
|  |  | Cases: 128 |  |  | 6 months after DRF surgery | <55 years: 34.1 (32.3 to 35.9)  55-64 years: 32.4 (27.8 to 37)  65-74 years: 31.2 (29.4 to 32.9)  >74 years: 27.7 (25.5 to 30) | NR |
| Crockett et al., [30] | Case series | 63 | Functional reach test | cm | 3 weeks | 32 (5.84) | N/A |
|  |  |  |  |  | 12 weeks | 34 (6.35) | NR |
|  |  |  |  |  | 26 weeks | 33.3 (6.35) | NR |
|  |  |  |  |  | 52 weeks | 31.2 (6.35) | NR |
| Mehta et al., [15] | Case series | 21 | Functional reach test | cm | 7 weeks | 37.2 (5.2) | N/A |
| Crockett et al., [17] | Case-control | Cases: 30 | Functional reach test component of BBS | cm | 6-24 months | 29.6 (7.7) | N/A |
| **BBS** | | | | | | | |
| Crockett et al., [17] | Case-control | Cases: 30 | BBS | ^‖^Score: 0-56 | 6-24 months | 53.9 (5.8) | N/A |
| Edwards et al., [18] | Case-control | Cases: 26 | BBS | ^‖^Score: 0-56 | 6-24 months | 51.8 (3.9) | N/A |
| **Biodex Balance System** | | | | | | | |
| Dewan et al., [32] |  |  | Biodex Balance System | Biodex stability index | 1-2 weeks | 50-64 years: 2.1 (1.2)  65-80 years: 2.3 (1.1) | N/A |
| Wong et al., [35] | RCT | 90 (per treatment group NR) | Biodex Balance System | Overall stability index | Baseline (6 weeks–3 months after DRF) | NR | N/A |
|  |  |  |  | Anteroposterior stability index |  |  |  |
|  |  |  |  | Medial/later stability index |  |  |  |
|  |  |  |  | Limits of stability |  |  |  |
|  |  |  |  | Overall stability index | 3 months after baseline | Significant improvement in int compared to con, p = 0.049 | NR |
|  |  |  |  | Anteroposterior stability index |  | NR |  |
|  |  |  |  | Medial/later stability index |  | Significant improvement in int compared to con, p = 0.046 |  |
|  |  |  |  | Limits of stability |  | Significant improvement in int compared to con, p = 0.049 |  |

Data are mean (standard deviation) unless otherwise stated; ^†^Only applies to instrumented measurements; ^‡^Time after distal radius fracture unless otherwise stated; ^§^Time after distal radius fracture not reported; ^¶^Only data for participants that completed follow-up assessment for this outcome reported; ^‖^Higher score better; BBS: Berg Balance Scale; CI: Confidence interval; cm: centimetre; cm/s: centimetres per second; Con: Control group; DRF: Distal radius fracture; Int: Intervention group; L: Left leg; max: Maximum; mm: millimetres; N/A: Not applicable; NR: Not reported; R: Right leg; RCT: Randomised controlled trial; s: Seconds; SD: Standard deviation
